# Meta-analysis identifies seven susceptibility loci involved in the atopic march

**DOI:** 10.1038/ncomms9804

**Published:** 2015-11-06

**Authors:** Ingo Marenholz, Jorge Esparza-Gordillo, Franz Rüschendorf, Anja Bauerfeind, David P. Strachan, Ben D. Spycher, Hansjörg Baurecht, Patricia Margaritte-Jeannin, Annika Sääf, Marjan Kerkhof, Markus Ege, Svetlana Baltic, Melanie C. Matheson, Jin Li, Sven Michel, Wei Q. Ang, Wendy McArdle, Andreas Arnold, Georg Homuth, Florence Demenais, Emmanuelle Bouzigon, Cilla Söderhäll, Göran Pershagen, Johan C. de Jongste, Dirkje S. Postma, Charlotte Braun-Fahrländer, Elisabeth Horak, Ludmila M. Ogorodova, Valery P. Puzyrev, Elena Yu Bragina, Thomas J. Hudson, Charles Morin, David L. Duffy, Guy B. Marks, Colin F. Robertson, Grant W. Montgomery, Bill Musk, Philip J. Thompson, Nicholas G. Martin, Alan James, Patrick Sleiman, Elina Toskala, Elke Rodriguez, Regina Fölster-Holst, Andre Franke, Wolfgang Lieb, Christian Gieger, Andrea Heinzmann, Ernst Rietschel, Thomas Keil, Sven Cichon, Markus M. Nöthen, Craig E. Pennell, Peter D. Sly, Carsten O. Schmidt, Anja Matanovic, Valentin Schneider, Matthias Heinig, Norbert Hübner, Patrick G. Holt, Susanne Lau, Michael Kabesch, Stefan Weidinger, Hakon Hakonarson, Manuel A. R. Ferreira, Catherine Laprise, Maxim B. Freidin, Jon Genuneit, Gerard H. Koppelman, Erik Melén, Marie- Hélène Dizier, A John Henderson, Young Ae Lee

**Affiliations:** 1 Max-Delbrück-Center (MDC) for Molecular Medicine, Berlin, Germany; 2 Clinic for Pediatric Allergy, Experimental and Clinical Research Center, Charité University Medical Center, Berlin, Germany; 3 Population Health Research Institute, St George's, University of London, London, UK; 4 Institute of Social and Preventive Medicine, University of Bern, Bern, Switzerland; 5 Department of Dermatology, Allergology, and Venerology, University Hospital Schleswig-Holstein, Campus Kiel, Kiel, Germany; 6 Inserm, UMR-946, F-75010 Paris, France; 7 Université Paris Diderot, Sorbonne Paris Cité, Institut Universitaire d'Hématologie, F-75007 Paris, France; 8 Institute of Environmental Medicine, Karolinska Institutet, Stockholm, Sweden; 9 University of Groningen, University Medical Center Groningen, GRIAC Research Institute, Groningen, The Netherlands; 10 Dr von Hauner Children's Hospital, Ludwig Maximilians University Munich, Member of the German Center for Lung Research (DZL), Munich, Germany; 11 Lung Institute of Western Australia, University of Western Australia, Perth, Western Australia, Australia; 12 Centre for Molecular, Environmental, Genetic and Analytic Epidemiology, University of Melbourne, Melbourne, Victoria, Australia; 13 Center for Applied Genomics, The Children's Hospital of Philadelphia, Philadelphia, Pennsylvania, USA; 14 Department of Pediatric Pneumology and Allergy, University Childreǹs Hospital Regensburg (KUNO), Regensburg, Germany; 15 School of Women and Infant's Health, University of Western Australia, Perth, Western Australia, Australia; 16 School of Social and Community Medicine, University of Bristol, Bristol, UK; 17 Clinic and Polyclinic of Dermatology, University Medicine Greifswald, Greifswald, Germany; 18 Department of Functional Genomics, Interfaculty Institute for Genetics and Functional Genomics, University Medicine and Ernst-Moritz-Arndt-University Greifswald, Greifswald, Germany; 19 Department of Biosciences and Nutrition, and Center for Innovative Medicine (CIMED), Karolinska Institutet, Stockholm, Sweden; 20 Department of Pediatrics, Pediatric Chest Center, Erasmus University Medical Center, Rotterdam, The Netherlands; 21 Department of Pulmonology and GRIAC Research Institute, University of Groningen, University Medical Center Groningen, Groningen, The Netherlands; 22 Swiss Tropical and Public Health Institute and the University of Basel, Basel, Switzerland; 23 Division of Pediatric Cardiology and Pulmonology, Department of Pediatrics, Innsbruck Medical University, Innsbruck, Austria; 24 Siberian State Medical University, Tomsk, Russia; 25 Research Institute of Medical Genetics, Tomsk, Russia; 26 Ontario Institute for Cancer Research, Toronto, Ontario, Canada; 27 Centre de santé et de services sociaux de Chicoutimi, Saguenay, Québec, Canada; 28 QIMR Berghofer Medical Research Institute, Brisbane, Queensland, Australia; 29 Woolcock Institute of Medical Research, University of Sydney, Sydney, New South Wales, Australia; 30 Department of Respiratory Medicine, Murdoch Children's Research Institute, Melbourne, Victoria, Australia; 31 Busselton Population Medical Research Foundation, Sir Charles Gairdner Hospital, Perth, Western Australia, Australia; 32 Department of Pediatrics, Perelman School of Medicine, University of Pennsylvania, Philadelphia, Pennsylvania, USA; 33 Department of Otolaryngology—Head and Neck Surgery, Temple University, School of Medicine, Philadelphia, Pennsylvania, USA; 34 Institute of Clinical Molecular Biology, Christian-Albrechts-University of Kiel, Kiel, Germany; 35 Institute of Epidemiology, Christian-Albrechts-University of Kiel, Kiel, Germany; 36 Research Unit of Molecular Epidemiology and Institute of Epidemiology II, Helmholtz Zentrum München, German Research Center for Environmental Health, Neuherberg, Germany; 37 University Children's Hospital, Albert Ludwigs University, Freiburg, Germany; 38 University Children's Hospital, University of Cologne, Cologne, Germany; 39 Institute of Social Medicine, Epidemiology and Health Economics, Charité University Medical Center, Berlin, Germany; 40 Institute for Clinical Epidemiology and Biometry, University of Würzburg, Würzburg, Germany; 41 Institute of Human Genetics, University of Bonn, Bonn, Germany; 42 Department of Genomics, Life & Brain Center, University of Bonn, Bonn, Germany; 43 Division of Medical Genetics, University Hospital Basel and Department of Biomedicine, University of Basel, Basel, Switzerland; 44 Institute of Neuroscience and Medicine (INM-1), Structural and Functional Organisation of the Brain, Genomic Imaging, Research Centre Jülich, Jülich, Germany; 45 Queensland Children's Medical Research Institute, University of Queensland, Brisbane, Queensland, Australia; 46 Institute for Community Medicine, Study of Health in Pomerania/KEF, University Medicine Greifswald, Greifswald, Germany; 47 Max Planck Institute for Molecular Genetics, Berlin, Germany; 48 Telethon Kids Institute, University of Western Australia, Perth, Western Australia, Australia; 49 Department of Pediatric Pneumology and Immunology, Charité University Medical Center, Berlin, Germany; 50 Université du Québec à Chicoutimi, Saguenay, Québec, Canada; 51 Institute of Epidemiology and Medical Biometry, Ulm University, Ulm, Germany; 52 Department of Pediatric Pulmonology and Pediatric Allergology, University of Groningen, University Medical Center Groningen, Beatrix Children's Hospital and GRIAC Research Institute, Groningen, The Netherlands; 53 Sachs' Children's Hospital, Stockholm, Sweden

## Abstract

Eczema often precedes the development of asthma in a disease course called the ‘atopic march'. To unravel the genes underlying this characteristic pattern of allergic disease, we conduct a multi-stage genome-wide association study on infantile eczema followed by childhood asthma in 12 populations including 2,428 cases and 17,034 controls. Here we report two novel loci specific for the combined eczema plus asthma phenotype, which are associated with allergic disease for the first time; rs9357733 located in *EFHC1* on chromosome 6p12.3 (OR 1.27; *P=*2.1 × 10^−8^) and rs993226 between *TMTC2* and *SLC6A15* on chromosome 12q21.3 (OR 1.58; *P=*5.3 × 10^−9^). Additional susceptibility loci identified at genome-wide significance are *FLG* (1q21.3), *IL4/KIF3A* (5q31.1), *AP5B1/OVOL1* (11q13.1), *C11orf30/LRRC32* (11q13.5) and *IKZF3* (17q21). We show that predominantly eczema loci increase the risk for the atopic march. Our findings suggest that eczema may play an important role in the development of asthma after eczema.

## Abstract (web summary)

The development of asthma following eczema is known as the atopic march. Here the authors conduct a GWAS on affected children and identify two novel loci associated with the disease phenotype.

## Graphical abstract

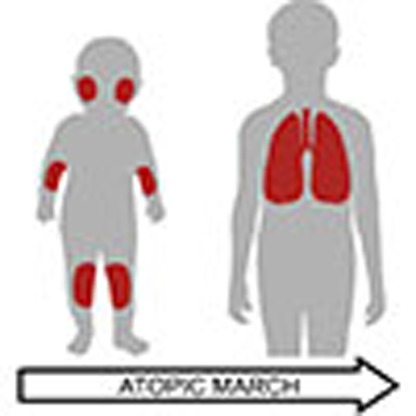

The atopic or allergic march describes the sequential progression of different allergic conditions frequently observed in children with IgE-antibody responses against common environmental allergens (atopy)^1^,^2^. Generally, eczema (atopic dermatitis) is the first clinical manifestation of the atopic march followed by asthma and/or allergic rhinitis. About 20–30% of infants with eczema undergo this unfavourable disease course, which is associated with severe and persistent allergic disease manifestations^3^,^4^.

In recent years, the concept of the atopic march has received increasing attention^5^,^6^,^7^. Multiple progression patterns have been discussed as allergic conditions may manifest in different orders^1^. A breakthrough in the understanding of the atopic march was the discovery of the filaggrin loss-of-function mutations that provided genetic evidence linking skin barrier deficiency to eczema and subsequent asthma development^8^,^9^.

We aimed to identify the genetic factors underlying the atopic march in a genome-wide association study (GWAS). We used the most common phenotype representing the atopic march, which is eczema followed by asthma^10^. In the discovery phase, six GWASs were included and another six populations were used for replication. Our meta-analysis identifies seven susceptibility loci at genome-wide significance of which two are associated with allergic disease for the first time. In addition, we find an overrepresentation of eczema loci among the atopic march loci. Deciphering the molecular determinants of the atopic march may point to novel therapeutic approaches to prevent or at least arrest the atopic march.

## Results

### Meta-GWAS of the discovery populations

To identify genes involved in the atopic march, we performed GWASs in six populations including 1,151 cases and 10,030 controls of European descent, and meta-analysed the results (Supplementary Fig. 1; Supplementary Table 1). We used a strict phenotype definition focusing on individuals with early-onset eczema (up to 3 years of age) and childhood asthma (up to 16 years of age; Supplementary Table 2). Association with disease was calculated by logistic regression using an additive allele-dosage model. For each population of the discovery set, more than two million single nucleotide polymorphisms (SNPs) imputed from the HapMap 2 Utah Residents with Northern and Western European Ancestry (CEU) reference panel were available.

**Figure 1:**
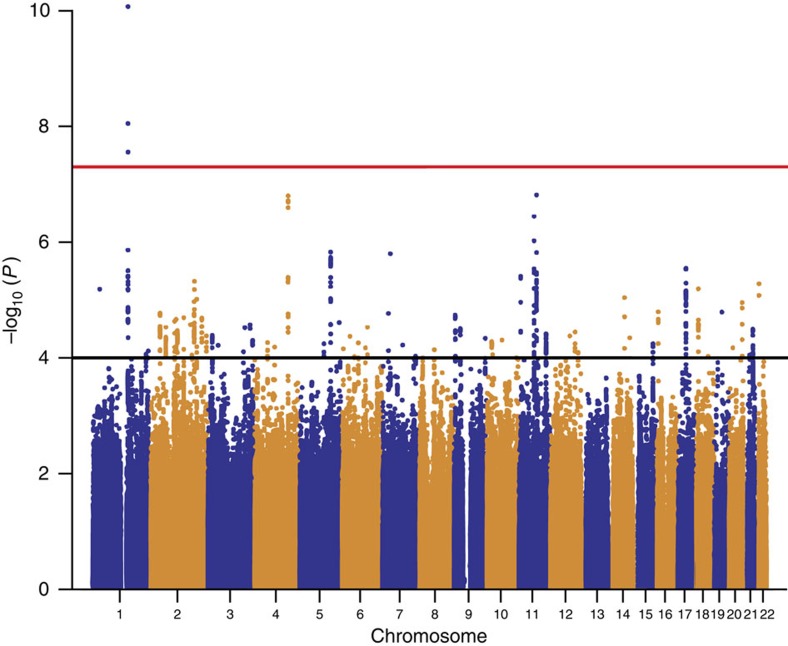
Association results of the meta-GWAS on the atopic march. Manhattan plot shows the *P* values over the chromosomal positions of all SNPs of the discovery set (1,151 cases versus 10,030 controls). Red and black lines indicate the thresholds for genome-wide significance (*P<*5 × 10^−8^) and for entering the replication phase (*P<*1 × 10^−4^), respectively.

More than 1.6 million SNPs passed the quality control criteria in all study populations and remained in the meta-analysis (see Methods section). There was little evidence for inflation of test statistics (*λ*_GC(meta)_=1.07, *λ*_1,000_=1.03 (ref. 11); Supplementary Fig. 2). Several SNPs within the epidermal differentiation complex (EDC) on chromosome 1q21.3 reached genome-wide significance (Fig. 1). We found strong correlation between the lead SNP rs12081541 (odds ratio (OR) 1.61; *P*=8.5 × 10^−11^), and a nearby loss-of-function mutation in the filaggrin gene (*FLG* R501X, D′=0.86, *r*^2^=0.18), a well-known risk factor for eczema and eczema-associated asthma^8^,^9^. After adjusting for *FLG*-null mutations in the study populations with available genotypes, the association signal in the EDC disappeared (Supplementary Fig. 3), indicating that there is no additional susceptibility locus within this region.

### Replication and combined meta-analysis

In total, 54 SNPs from regions with moderate association in the discovery set (1 × 10^−4^>*P>*5 × 10^−8^; Fig. 1) were subjected to replication in five independent study populations including 864 atopic march cases and 5,346 controls of European descent (replication set 1; Supplementary Table 1). When multiple associated SNPs were located in the same region, we selected the best SNP per 1-Mb window. We meta-analysed the results of the study populations in replication set 1 (Supplementary Table 3) and, in addition, performed a combined meta-analysis including the discovery set. In the combined meta-analysis, four additional susceptibility loci were associated with the atopic march at genome-wide significance (*P<*5 × 10^−8^; Table 1).

The susceptibility loci *IL4*/*KIF3A* on chromosome 5 (rs17690965; OR 1.24; *P=*4.5 × 10^−8^) and *OVOL1* on chromosome 11 (rs479844; OR 1.25; *P=*3.6 × 10^−10^) were previously identified in a GWAS on eczema (Supplementary Table 4)^12^. In addition, the association signal near *C11orf30/LRRC32* on chromosome 11 (rs2155219; OR 1.33; *P=*1.8 × 10^−15^) confirmed a susceptibility locus for eczema and eczema-associated asthma^13^,^14^. Finally, the chromosomal region around rs10445308 (OR 1.22; *P=*4.7 × 10^−8^), which is located in an intron of *IKZF3* on chromosome 17 was found previously in studies on childhood asthma^15^,^16^,^17^, self-reported allergy^18^ and asthma plus hay fever (Supplementary Table 5)^19^. Regional association plots of the atopic march susceptibility regions in the populations of the discovery set are depicted in Supplementary Fig. 4.

Importantly, two novel susceptibility loci for the atopic march were identified: both variants (rs9357733 and rs993226) were significantly associated in replication set 1 after correction for multiple testing (*P*<0.05/54 (9.3 × 10^−4^); Supplementary Table 3) but did not reach genome-wide significance in the combined analysis. Both SNPs were therefore genotyped in a second independent replication set of 413 cases and 1,658 controls from Europe (replication set 2), and reached genome-wide significance in the combined meta-analysis including all data sets (Table 1).

### Characterization of the two novel atopic march loci

As a preliminary step towards understanding the functional basis of the novel atopic march loci, we evaluated their association with gene expression in lymphoblastoid cell lines of 373 European individuals from the 1000 Genomes Project^20^, reviewed their functional annotations in ENCODE Consortium ( http://genome.ucsc.edu/ENCODE/)^21^ and Roadmap Epigenomics Consortium data ( http://www.roadmapepigenomics.org/)^22^, and surveyed relevant mouse models ( http://www.informatics.jax.org/phenotypes.shtml)^23^.

On chromosome 6p12.3, rs9357733 was associated with the atopic march (OR 1.27; *P=*2.1 × 10^−8^; Table 1) with consistent risk allele and effect size across all study populations (Supplementary Fig. 5). It is located 20 Mb away from the established risk variants for eczema and for asthma on 6p21.3 and not in linkage disequilibrium with them. The linkage disequilibrium block around rs9357733 contains the genes encoding the progestin and adipoQ receptor family member VIII (*PAQR8*), the EF-hand domain (C-terminal)-containing protein 1 (*EFHC1*) and the translocation associated membrane protein 2 (*TRAM2*; Supplementary Fig. 4). Strongest association was observed in intron 2 of *EFHC1*, which is ubiquitously expressed ( http://fantom.gsc.riken.jp/zenbu/)^24^. We used SNAP (SNP Annotation and Proxy Search; http://www.broadinstitute.org/mpg/snap/ldsearch.php)^25^ to screen for functional SNPs in linkage disequilibrium with rs9357733 and identified rs3804508 (*r*^2^=1) located in a predicted enhancer (Supplementary Fig. 6). The corresponding enhancer marks were predominantly found in cells derived from target organs of allergic disease such as skin keratinocytes, skin fibroblasts and lung fibroblasts, and were absent in immune cells.

A second novel variant associated with the atopic march, rs993226 (OR 1.58; *P=*5.3 × 10^−9^; Table 1), is located on chromosome 12q21.3, 1.36 Mb distal of transmembrane and tetratricopeptide repeat containing 2 (*TMTC2*) and 370 kb proximal of solute carrier family 6 (neurotransmitter transporter), member 15 (*SLC6A15*; Supplementary Fig. 4). Little is known about the more distant gene *TMT2*, which encodes a membrane protein of the endoplasmatic reticulum involved in calcium homeostasis^26^. *SLC6A15* encodes a Na^+^/Cl^−^-dependent membrane transporter for neutral amino acids, B(0)AT2, which exhibits a specific gene expression pattern predominantly in cells derived from skin, respiratory tract and brain ( http://fantom.gsc.riken.jp/zenbu/)^24^. However, there was no evidence from expression quantitative trait locus (eQTL) or epigenetic data for an involvement of rs993226 in the regulation of these genes (Supplementary Table 6).

### The role of eczema loci and asthma loci in the atopic march

**Figure 2:**
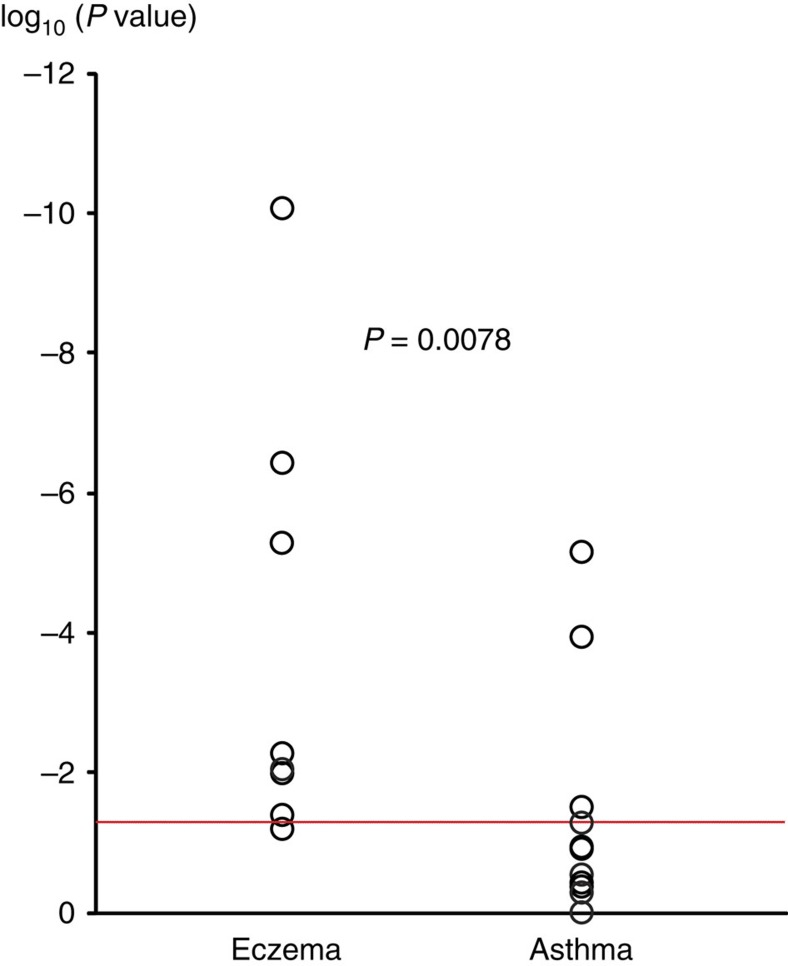
Ranking of previous eczema and asthma GWAS hits in the atopic march discovery set. Susceptibility loci identified in previous GWAS on eczema or on asthma at genome-wide significance (*P<*5 × 10^−8^) are shown with their *P* values in the discovery phase (circles). The red line indicates the threshold for nominally significant replication (*P<*0.05). Susceptibility loci previously associated with both traits were excluded as were loci identified only in populations of non-European descent. Two-sided *P* value of the Mann-Whitney *U*-test ( http://vassarstats.net/utest.html) is reported.

Next, we evaluated whether previously reported susceptibility loci for eczema or asthma were associated with the atopic march in our discovery meta-analysis. From the catalog of published GWASs^27^, we selected all SNPs which were associated with asthma or eczema at genome-wide significance level (Supplementary Tables 4 and 5), and examined association in our discovery set. All five GWAS loci previously associated with both traits (*IL6R*, *IL1RL1*/*IL18R1*, *RAD50*, human leukocyte antigen (HLA) region and *C11orf30*) replicated at *P<*0.001 in our data set. For the European susceptibility loci that were previously identified only in studies on eczema, we found the same risk alleles in our study; seven out of eight were associated with the atopic march (*P<*0.05; Supplementary Table 4). In contrast, only 3 out of 12 European loci identified only in GWAS on asthma were associated at *P<*0.05 (Supplementary Table 5). Moreover, ranking the known risk variants, which were specific for asthma or eczema according to their statistical significance in the discovery meta-analysis yielded a significant enrichment of eczema loci among the SNPs associated with the atopic march (*P=*0.0078; Fig. 2).

### Subphenotype analysis for the atopic march loci

Since we studied the combined phenotype of eczema plus asthma, we investigated whether the identified associations were driven by either eczema or asthma. We performed this analysis in the large population-based Avon Longitudinal Study of Parents and Children (ALSPAC) birth cohort (*n*=7,829 genotyped individuals). The strengths of ALSPAC are longitudinal data on eczema and on asthma throughout childhood. This allowed us to define all three phenotypes (atopic march, eczema alone and asthma alone) in a single, unselected cohort and to use identical controls for all three comparisons. In line with the meta-analysis, six of seven loci were significantly associated with the atopic march (*P<*0.05) and only one locus just failed significance (Table 2). The four loci originally discovered through GWAS on eczema (*FLG*, *IL4*/*KIF3A*, *OVOL1* and *C11orf30*/*LRRC32*) did not reveal an effect on asthma alone. Compared with eczema alone, the excess risk of these loci on the atopic march was not significant (Supplementary Tables 7 and 8) although a trend for *FLG*-null mutations and the *C11orf30*/*LRRC32* locus was observed. For both of these loci, an effect on disease progression from eczema to asthma has previously been demonstrated^9^,^13^, suggesting that our current analysis may have lacked power to detect such an effect. The previously identified asthma locus *IKZF3* revealed a different association pattern with a stronger effect on asthma than on the atopic march. Interestingly, the two new loci conferred risk for the atopic march but not for eczema alone or asthma alone (Table 2).

### Assessment of a potential selection bias

All cases included in this meta-analysis of the atopic march had participated in previous GWAS on eczema or asthma. Therefore, we had to rule out that the observed excess of eczema susceptibility loci was due to an overrepresentation of eczema studies. The inclusion of study populations that were originally recruited through eczema, for example, may favour the discovery of eczema loci. However, only two study populations were recruited through eczema (*n*=402 atopic march cases), while six were unselected (*n*=1,137 atopic march cases) and three were recruited through asthma (*n*=476 atopic march cases; Supplementary Table 9). In addition, we determined to what extent our samples contributed to the discovery of eczema loci and asthma loci in previous GWAS. Considering the cases in previous eczema and asthma GWAS, on an average only 10.8 and 11.0%, respectively, were included in the current atopic march study (Supplementary Table 10). It is therefore unlikely that the prominent role of eczema loci in our study was due to a selection bias.

## Discussion

The atopic march describes disease progression from infantile eczema to allergic airway disease in childhood and is associated with severe and persistent allergic disease manifestations. Here, we report the first GWAS on the atopic march. We identified seven genome-wide significant loci, of which two susceptibility loci, in *EFHC1* on chromosome 6p12.3 and between *TMTC2* and *SLC6A15* on chromosome 12q21.3 were associated with allergic disease for the first time.

Coding variants in *EFHC1* have been implicated in juvenile forms of epilepsy^28^,^29^,^30^,^31^,^32^. In a knockout-mouse model, reduced beating frequency of the cilia of ependymal cells in the brain due to lack of *Efhc1/myoclonin1* was identified as potential disease mechanism^33^. Interestingly, *Efhc1* was also detected in motile cilia of tracheal epithelium in mice^34^. The mucociliary epithelium is important for the defence against inhaled pathogens and allergens and ciliary dysfunction is a feature of severe asthma^35^. Association between epilepsies and allergic diseases in childhood has recently been demonstrated^36^, which may point to a common molecular mechanism or to pleiotropic effects.

SNPs in *SLC6A15* were previously associated with depression, stress-induced cortisol secretion and obesity-related phenotypes^37^,^38^,^39^. A potential functional role of *SLC6A15* in the atopic march is suggested by a recent study demonstrating selective inhibition of B(0)AT2 by the histamine H1 receptor antagonist loratadine^40^, which is clinically used for the treatment of allergic disease. Further studies will be necessary to elucidate a potential functional role of this genomic region. Interestingly, both novel loci revealed no effect on eczema alone or asthma alone. In agreement with these results, they were not found in previous GWAS on either trait and may represent novel disease mechanisms reinforcing the existence of a specific atopic march phenotype.

In our study, GWAS loci for eczema ranked significantly higher than asthma loci. In addition, we observed a stronger effect of the atopic march loci on eczema alone than on asthma alone for four of the five established allergic disease loci. These results suggest that genes triggering eczema are the predominant driver of the atopic march. In contrast, the majority of the GWAS loci for asthma seem to be more involved in an eczema-independent phenotype, supporting genetic heterogeneity in asthma susceptibility. This finding highlights the role of the skin as the interface between host and environment in shaping local and systemic immune responses that induce chronic inflammation and may affect distant organs in the host^41^. In mice, it has been demonstrated that enhanced transcutaneous penetration of allergens promotes the formation of allergen-specific IgE antibodies^42^,^43^ and bronchial hyperresponsiveness^43^. In humans, a strong association of *FLG*-null mutations with eczema, with disease progression to asthma, but not with asthma alone is well documented^8^,^9^. This finding provided the first genetic link between the loss of a skin-specific-protein, cutaneous barrier deficiency, eczema and subsequent asthma development. Furthermore, the importance of infantile eczema in subsequent asthma susceptibility is underlined by epidemiological data in the ALSPAC cohort indicating that early eczema confers a 4.3-fold increase in asthma risk (OR 4.33; 95% confidence interval (CI) 3.72–5.01, *P*<0.0001). A causal relationship between eczema and this asthma subtype would have important implications for interventional strategies. Since no cure for asthma exists, one might speculate that modulation of skin integrity early in infancy could be an effective strategy to prevent not only eczema but also the atopic march in a subset of patients. Recently, skin emollient therapy in neonates yielded promising results regarding eczema prevention^5^,^44^,^45^. However, a long-term effect on asthma in these children still remains to be demonstrated. Epidemiological studies demonstrating an increased asthma risk in children with early eczema, clearly support the concept of the atopic march and the phenotype under study^46^,^47^,^48^,^49^. In the largest study on the progression of multiple allergic diseases, 60.7% of allergic children developed eczema first^10^. Of all children with eczema, 86% had it as their first allergic condition, indicating that it is uncommon to develop eczema after asthma or allergic rhinitis. The development of all three conditions, eczema, asthma and allergic rhinitis was unusual occurring in only 2.3% of all children. Therefore, defining the atopic march based on all three allergic diseases may underestimate its prevalence^50^. Among all children with multiple allergic diseases, the single largest group were those with eczema followed by asthma (38.3%), the phenotype used in our current study.

Other atopic march patterns exist as allergic conditions may occur in different orders. In addition, since eczema and asthma are common diseases, they may also co-manifest coincidentally. Thus, the identification of known eczema and asthma loci in this study may reflect the underlying association with each disease independently. In addition, the role of less prevalent allergic manifestations in the atopic march still needs to be resolved. For food allergy, mouse models point to a potential involvement in the atopic march^51^,^52^, but robust data from longitudinal cohorts are sparse^53^,^54^,^55^,^56^.

Some limitations of our study need to be discussed. Although most of our study populations selected strict controls by the absence of eczema and asthma, others used population-based controls with unknown disease status. Accordingly, the presence of affected individuals among controls may have decreased the power of these studies. In addition, we did not use atopy status in our meta-analysis due to missing data in some populations. Where available, atopy data were very heterogeneous between studies regarding age at testing, assay used, number of data points and allergens tested. The inclusion of atopic controls may have reduced the power to detect atopy-related genes involved in the atopic march. However, homogeneous effects of the atopic march loci across our study populations (Supplementary Fig. 5) using selected and unselected (population-based) controls pointed to a minor effect of the selection process.

We did not investigate allergic rhinitis in our study because reliable phenotype data were not available for all study populations. As a consequence, our results could be biased towards genes involved in eczema or asthma rather than allergic rhinitis. However, the only significant locus identified in GWAS on allergic rhinitis, *C11orf30*/*LRRC32* (ref. 57), is a known risk factor for eczema-associated asthma and hay fever^13^, suggesting that similar mechanisms may be involved in the atopic march from eczema to asthma and from eczema to allergic rhinitis.

For the phenotype definition, we required early eczema to have manifested by 3 years of age and childhood asthma by 16 years. However, in several populations phenotype data were not available for the complete time periods (Supplementary Table 2). Thus, those individuals who developed eczema and asthma within the missing time window may have been lost as cases. Their misclassification as controls is unlikely because this would require that both conditions, eczema and asthma, manifested within the respective missing period. In addition, a recall bias in retrospectively collected data can give rise to a loss of cases. Although most studies relied on actual or reported doctor's diagnoses, a few studies used a parental report of disease symptoms. Again, a loss of power can be expected, but case misclassifications requiring false reports of both eczema and asthma in the same individual are unlikely.

Since we used a strict atopic march definition of early-onset eczema and childhood asthma, a relatively small number of cases fulfilled our case definition. Using a wider definition of the atopic march (defined as eczema and asthma at any age up to 16 years) nearly doubled the number of cases in the discovery set and in the first replication to 2,075 and 1,591 cases, respectively (Supplementary Table 1). Applying the same quality control criteria and selection thresholds as in the strict definition, identified only five of the seven loci without evidence of additional susceptibility regions (Supplementary Table 11). Thus, our study demonstrates that the strict definition of a severe phenotype is a successful approach for studying complex diseases.

In summary, our study revealed two novel loci, which are specific for the atopic march phenotype. In addition, we found loci that were also associated with eczema alone or asthma alone, which may reflect heterogeneity of disease mechanisms underlying the atopic march. A strong contribution of eczema genes to the atopic march suggests that the development of eczema may play an important role in this unfavourable disease course.

## Methods

### Phenotype definition

The term ‘atopic march' refers to the sequential manifestations of different allergic diseases such as eczema, asthma and rhinitis^58^. Typically, eczema is the first allergic disease in infancy, followed by asthma and/or rhinitis in childhood^10^,^48^,^59^,^60^. More than 20% of the children with eczema develop asthma, making this the largest group among children with multiple allergic conditions^10^. Since complete longitudinal data were available only for a few study populations, we aimed to approximate the temporal component of the atopic march by using eczema up to the age of 3 years and asthma up to the age of 16 years (strict definition). For a total of 12 study populations, six of the discovery set and six of the replication set, the corresponding phenotype data were available (Supplementary Table 1). Another four study populations had no data on early eczema available. They were included in a separate analysis using a wider definition of the atopic march (childhood eczema plus asthma up to the age of 16 years).

Controls were selected for the absence of eczema and asthma if phenotype data were available. Four data sets included unselected, population-based controls. The phenotype characteristics of each study population are summarized in Supplementary Table 2. All but one study population of the discovery set had participated in the previous GWAS on asthma performed by the GABRIEL consortium^61^. All study populations were of European ancestry. Informed consent was obtained from all participants or their legal guardians, and all studies were approved by the local ethics committees as indicated in the Supplementary Methods.

### Genotyping and imputation

Genotyping and imputation of the study populations was performed in the context of the GABRIEL consortium or according to the methods described there^61^, if not otherwise stated. Briefly, after genotyping, an ancestry analysis was carried out using EIGENSTRAT2.0 (ref. 62) and putative non-European samples were eliminated from subsequent analyses. Genotyped SNPs for imputation had to fulfil the following quality control criteria: (1) genotype missing rate <3% in both cases and controls; (2) minor allele frequency ≥1% in controls; and (3) consistency with Hardy-Weinberg equilibrium by a one degree-of-freedom goodness-of-fit test in unselected controls (for case–control study populations) or in the whole population before case–control selection (for study populations derived from population-based cohorts), respectively (*P>*1 × 10^−4^). Imputation was carried out with MACH^63^ or IMPUTE^64^ using Hapmap 2 CEU SNPs (release 22, NCBI build 36) as reference.

### Association analyses

In each study population, association between SNPs and disease was analysed by logistic regression using an additive model for genotype dosage. Imputed SNPs with a quality score of *r*^2^<0.8 were filtered out. For data sets comprising unrelated and related cases and controls, we used a logistic regression model with robust sandwich estimation of the variance^65^ as implemented in the Stata logit function to model clustering of family genotypes. A detailed description of the study populations, including phenotype characteristics and genotyping platforms, and software used for imputation and logistic regression analysis is provided in the Supplementary File.

### Meta-analyses

Meta-analyses were carried out with METAL^66^ using the inverse variance fixed effects model. Combined ORs and 95% CIs were calculated. All SNPs with missing data in any of the study populations of the discovery set were excluded. For SNPs with a minor allele frequency of <5%, a quality score of *r*^2^≥0.9 was required in all data sets, for all other SNPs one data set with *r*^2^<0.9 was allowed. In addition, we tested for heterogeneity between cohorts using Cochran's Q statistic and excluded loci with evidence for effect heterogeneity (*P<*0.05). The genomic inflation factor was calculated for each cohort and for the combined analysis. We applied a threshold of *P<*5 × 10^−8^ to declare an effect as genome-wide significant. SNPs with *P<*1 × 10^−4^ in the discovery set meta-analysis were selected for replication. When multiple associated SNPs were located in the same region, we selected the best SNP per 1-Mb window. In total, 54 SNPs were selected and analysed using data from five additional GWAS sets. Two of these SNPs, which were significant after correcting for the number of SNPs in the replication set (*P<*0.05/54=*P<*9.3 × 10^−4^; Bonferroni correction), were genotyped in one more study population (Supplementary Fig. 1). Association analyses were again performed for each replication study separately using a logistic regression model. A meta-analysis was conducted for the replication set and for all studies combined as described above. We applied a genome-wide significance threshold of *P<*5 × 10^−8^ in the combined meta-analysis and tested for overall heterogeneity of the discovery and replication studies using the Cochran's Q statistic. Within the EDC in chromosomal region 1q21.3, we performed an association analysis adjusted for the effects of the most common mutations in the filaggrin gene (*FLG*), 2282del4 and R501X. *FLG* genotypes were available in ALSPAC, BAMSE, PIAMA and in a subset of the German samples with genome-wide data. The combined *FLG* mutation status was included as a covariate in the logistic regression analysis using an additive model.

### Genotyping of the *FLG* mutations R501X and 2282del4

The two most frequent *FLG*-null mutations in Europeans, R501X and 2282del4, were genotyped in ALSPAC^67^, the Children/Barn, Allergy, Milieu, Stockholm, an Epidemiological survey Study (BAMSE)^68^, the Prevention and Incidence of Asthma and Mite Allergy Study (PIAMA)^69^ and in the German set comprising the cases of the German GWAS and controls of the Population Genetics (POPGEN)/Cooperative health research in the region of Augsburg (KORA) studies^14^ as originally described^8^,^70^.

### Subphenotype analysis in the ALSPAC birth cohort

We analysed the association of the atopic march, eczema alone and asthma alone in the population-based birth cohort ALSPAC. For atopic march cases (*n*=563) and for controls (*n*=2,293), we used the same definition as in the present GWAS. For the eczema alone analysis, cases had to fulfil the early eczema definition and have a negative report on asthma (*n*=494). For the asthma alone analysis, cases had to fulfil the childhood asthma definition and have a negative report on eczema (*n*=587). In addition, we performed case-only analyses for the atopic march cases versus eczema alone and versus asthma alone. Effect sizes and CIs were assessed by logistic regression using PLINK.

Applying the same phenotype definitions, we assessed the effect of infantile eczema on childhood asthma by using a chi-square test.

## Additional information

**How to cite this article:** Marenholz, I. *et al.* Meta-analysis identifies seven susceptibility loci involved in the atopic march. *Nat. Commun.* 6:8804 doi: 10.1038/ncomms9804 (2015).

## Supplementary Material

Supplementary Information Supplementary Figures 1-5, Supplementary Tables 1-11, Supplementary Notes, Supplementary Methods and Supplementary References.

## Figures and Tables

**Table 1:** Association results of susceptibility loci associated with the atopic march.

SNP ID*	**Chr**	**Position**†	**EA**	**AA**	**Set**‡	**AF**	**OR**	**95% CI**	***P*** **value**	**Genes**
rs12081541§ (i)	1	150707990	c	t	D	0.091	1.61	1.41–1.89	8.5 × 10^−11^	*CRNN/LCE5A (FLG)*
rs17690965 (i)	5	132058566	c	g	D	0.259	1.28	1.16–1.42	1.5 × 10^−6^	*IL4/KIF3A*
					R1	0.271	1.18	1.05–1.32	4.8 × 10^−3^	
					Combined	0.264	1.24	1.15–1.33	4.5 × 10^−8^	
rs9357733 (i)	6	52400095	a	g	D	0.815	1.29	1.13–1.46	9.5 × 10^−5^	*EFHC1*
					R1	0.807	1.27	1.11–1.45	5.1 × 10^−4^	
					R2	0.792	1.22	1.01–1.47	0.038	
					Combined	0.808	1.27	1.17–1.38	2.1 × 10^−8^	
rs479844 (g)	11	65308533	g	a	D	0.558	1.28	1.16–1.41	3.6 × 10^−7^	*AP5B1/OVOL1*
					R1	0.563	1.22	1.25–1.54	2.0 × 10^−4^	
					Combined	0.560	1.25	1.24–1.43	3.6 × 10^−10^	
rs2155219 (i)	11	75976842	t	g	D	0.481	1.28	1.17–1.41	1.5 × 10^−7^	*C11orf30/LRRC32*
					R1	0.494	1.39	1.25–1.54	1.2 × 10^−9^	
					Combined	0.487	1.33	1.24–1.43	1.8 × 10^−15^	
rs993226 (g)	12	83410704	g	t	D	0.037	1.58	1.27–1.96	4.2 × 10^−5^	*SLC6A15/TMTC2*
					R1	0.033	1.59	1.23–2.04	4.0 × 10^−4^	
					R2	0.027	1.57	1.05–2.35	0.028	
					Combined	0.034	1.58	1.35–1.84	5.3 × 10^−9^	
rs10445308 (i)	17	35191573	c	t	D	0.530	1.25	1.14–1.37	2.9 × 10^−6^	*IKZF3*
					R1	0.528	1.18	1.05–1.30	2.9 × 10^−3^	
					Combined	0.529	1.22	1.14–1.30	4.7 × 10^−8^	

AA, alternative allele; AF, effect allele frequency; EA, effect allele; CI, confidence interval; OR, odds ratio.

^*^(i), imputed SNP; (g), genotyped SNP.

^†^Genomic positions were based on human genome reference NCBI Build 36.3.

^‡^D, discovery set; R1, replication set 1; R2, replication set 2; combined, combined analysis of D and R1 and of D, R1 and R2 (rs9357733 and rs993226 only), respectively.

^§^rs12081541 represents the known *FLG* risk locus and was not subjected to replication.

**Table 2:** Association of the atopic march loci with eczema alone and asthma alone in the ALSPAC cohort.

**SNP ID**	**Chr**	**Locus**	Original discovery*	**EA**	**AA**	**Atopic march**			**Eczema, no asthma**			**Asthma, no eczema**		
						**OR**	**95% CI**	***P*** **value**	**OR**	**95% CI**	***P*** **value**	**OR**	**95% CI**	***P*** **value**
rs9357733	6	*EFHC1*	Atopic march	a	g	1.27	1.07–1.52	0.0072	0.98	0.83–1.17	0.84	0.97	0.82–1.14	0.71
rs993226	12	*SLC6A15/TMTC2*	Atopic march	g	t	1.43	1.06–1.94	0.020	0.82	0.55–1.21	0.31	0.77	0.53–1.13	0.18
*FLG* combined†	1	*FLG*	Eczema	mut	wt	2.89	2.12–3.92	1.2 × 10^−11^	1.99	1.42–2.80	7.8 × 10^−5^	0.79	0.51–1.23	0.29
rs17690965	5	*IL4/KIF3A*	Eczema	c	g	1.18	1.02–1.37	0.025	1.12	0.96–1.31	0.15	1.03	0.89–1.19	0.72
rs479844	11	*OVOL1*	Eczema	g	a	1.24	1.08–1.42	0.0019	1.24	1.07–1.43	0.0032	1.00	0.87–1.14	0.95
rs2155219	11	*C11orf30/LRRC32*	Eczema	t	g	1.23	1.08–1.39	0.0016	1.12	0.98–1.28	0.097	1.05	0.92–1.19	0.49
rs10445308	17	*IKZF3*	Asthma	c	t	1.13	0.99–1.29	0.066	0.93	0.81–1.06	0.27	1.30	1.14–1.48	6.5 × 10^−5^

AA, alternative allele; EA, effect allele; CI, confidence interval; OR, odds ratio.

^*^Allergic phenotype first associated with SNP.

^†^ORs for *FLG* combined were estimated based on the two most frequent disease-causing mutations *FLG* R501X and *FLG* 2282del4; mut, mutated allele; wt, wild-type allele.
