# Supplementary material for: Meta-analysis identifies seven susceptibility loci involved in the atopic march
Source: Nat Commun. 2015 Nov 6;6:8804. doi: 10.1038/ncomms9804 (PMC4667629; doi:10.1038/ncomms9804)
Supplement: Supplementary Information — Supplementary Figures 1-5, Supplementary Tables 1-11, Supplementary Notes, Supplementary Methods and Supplementary References. [file ncomms9804-s1.pdf]

Supplementary Information

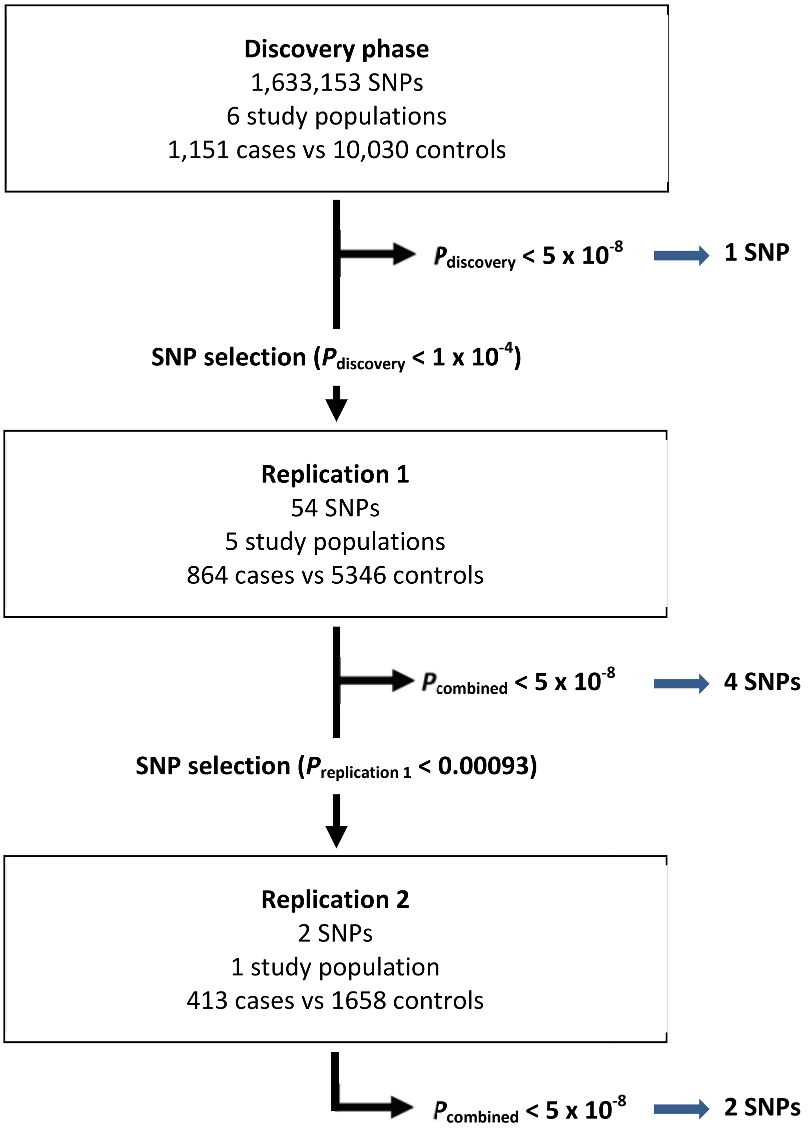

Supplementary Figure 1. Study design of the meta-GWAS on the atopic march.

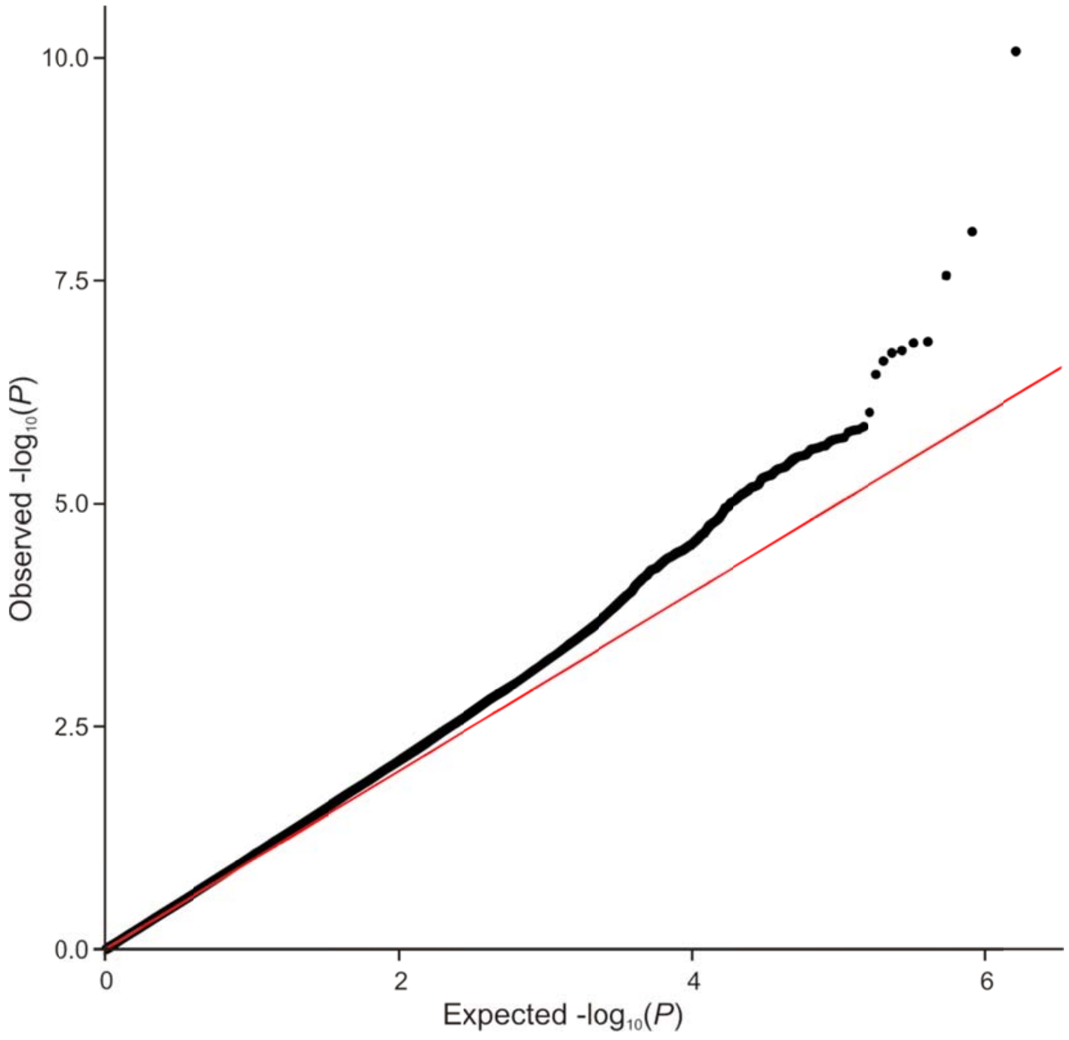

Supplementary Figure 2. Quantile-quantile plot of *P* values for the test statistics in the meta-GWAS. Horizontal and vertical axes show expected *P* values under a null distribution and observed *P* values, respectively.

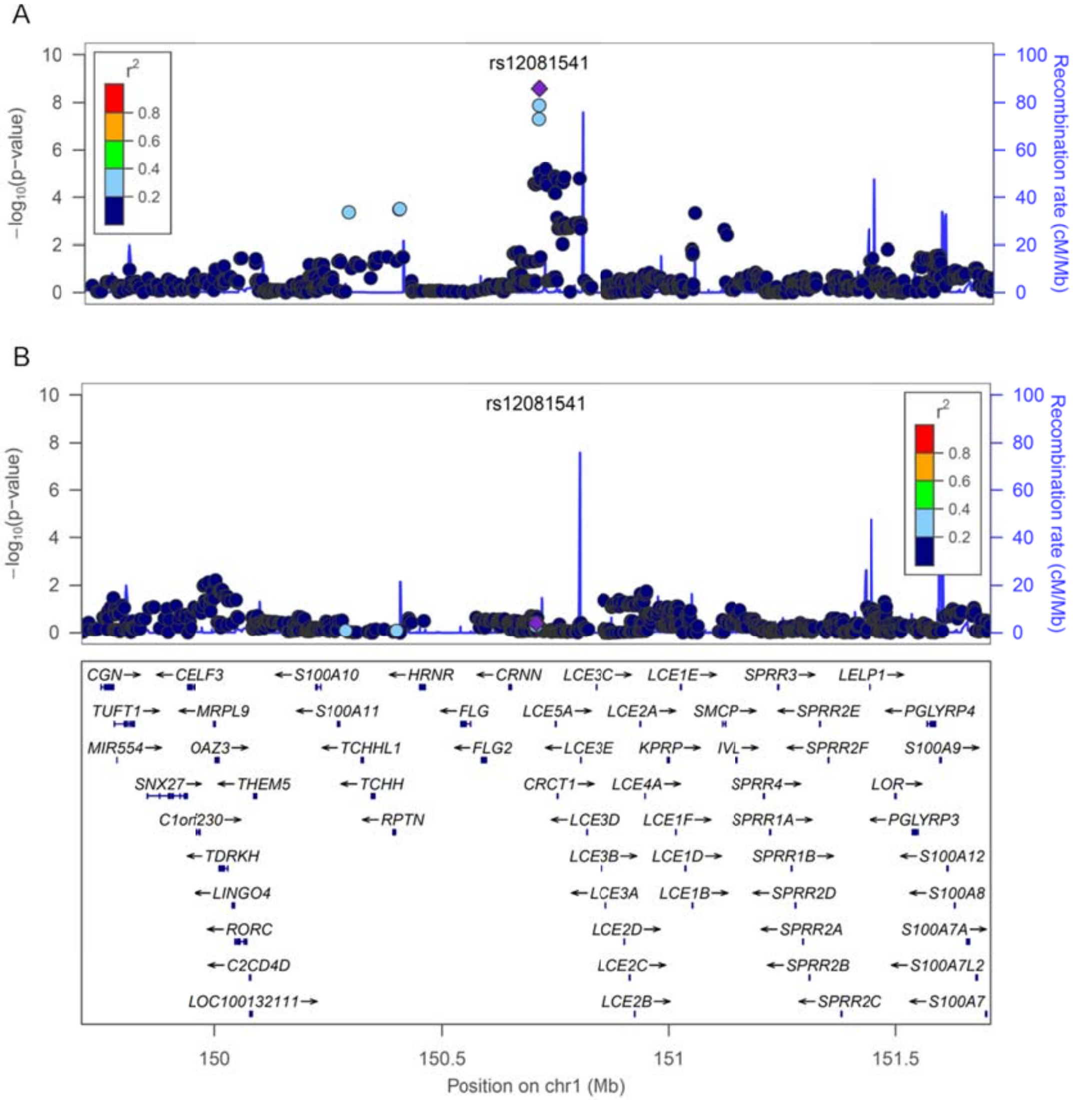

Supplementary Figure 3. Association results on chromosome 1q21.3 before (A) and after (B) adjusting for the effects of the two most common loss-of-function mutations in *FLG*, 2282del4 and R501X. Genomic position (x-axis) including the annotated genes and *P* values (y-axis) of the SNPs under study (dots) are indicated. Dots are colored according to the extent of LD (measured by r^2) between the respective SNP and the lead SNP (purple) in the region. Blue peaks represent recombination rates (y-axis). *FLG* genotypes were available from ALSPAC, BAMSE, PIAMA, and from a subset of the German samples. Plots were generated with LocusZoom (http://csg.sph.umich.edu/locuszoom/).[1]

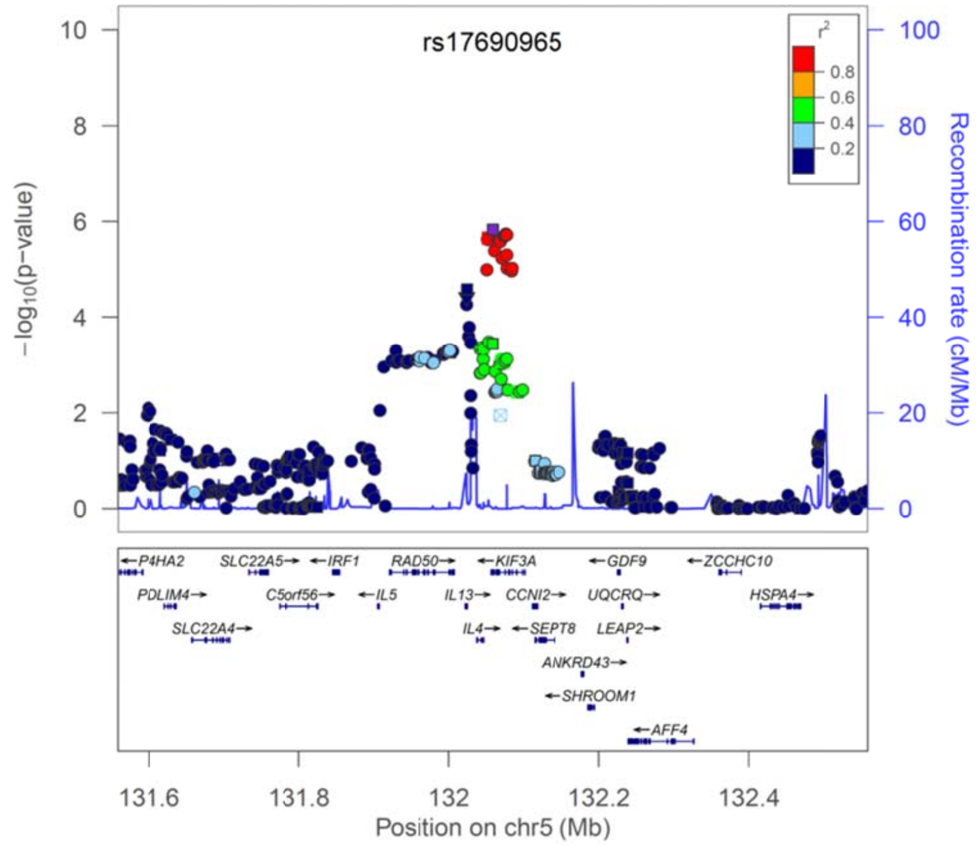
rs17690965
r2
0.8
0.6
0.4
0.2
−log10(p−value)
Recombination rate (cM/Mb)
P4HA2
SLC22A5
IRF1
RAD50
KIF3A
GDF9
ZCCHC10
PDLIM4
C5orf56
IL5
IL13
CCNI2
UQCRQ
HSPA4
SLC22A4
IL4
SEPT8
LEAP2
ANKRD43
SHROOM1
AFF4
131.6
131.8
132
132.2
132.4
Position on chr5 (Mb)

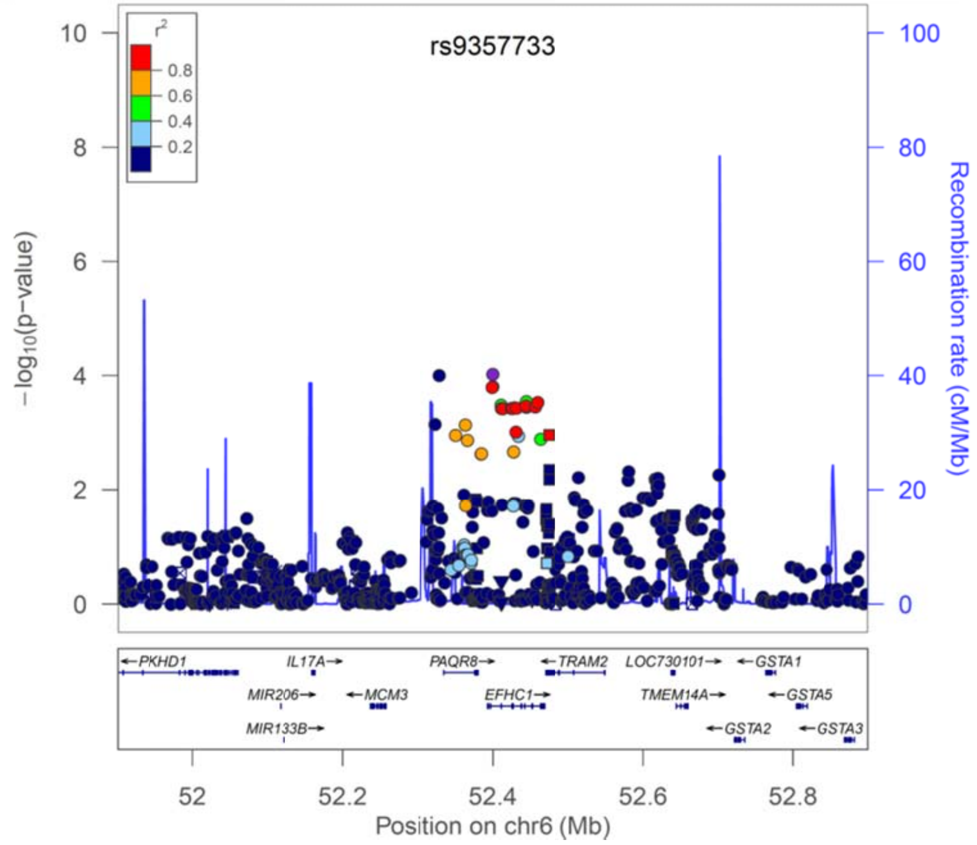
rs9357733
r2
0.8
0.6
0.4
0.2
−log10(p−value)
Recombination rate (cM/Mb)
PKHD1
IL17A
PAQR8
TRAM2
LOC730101
GSTA1
MIR206
MCM3
EFHC1
TMEM14A
GSTA5
MIR133B
GSTA2
GSTA3
52
52.2
52.4
52.6
52.8
Position on chr6 (Mb)

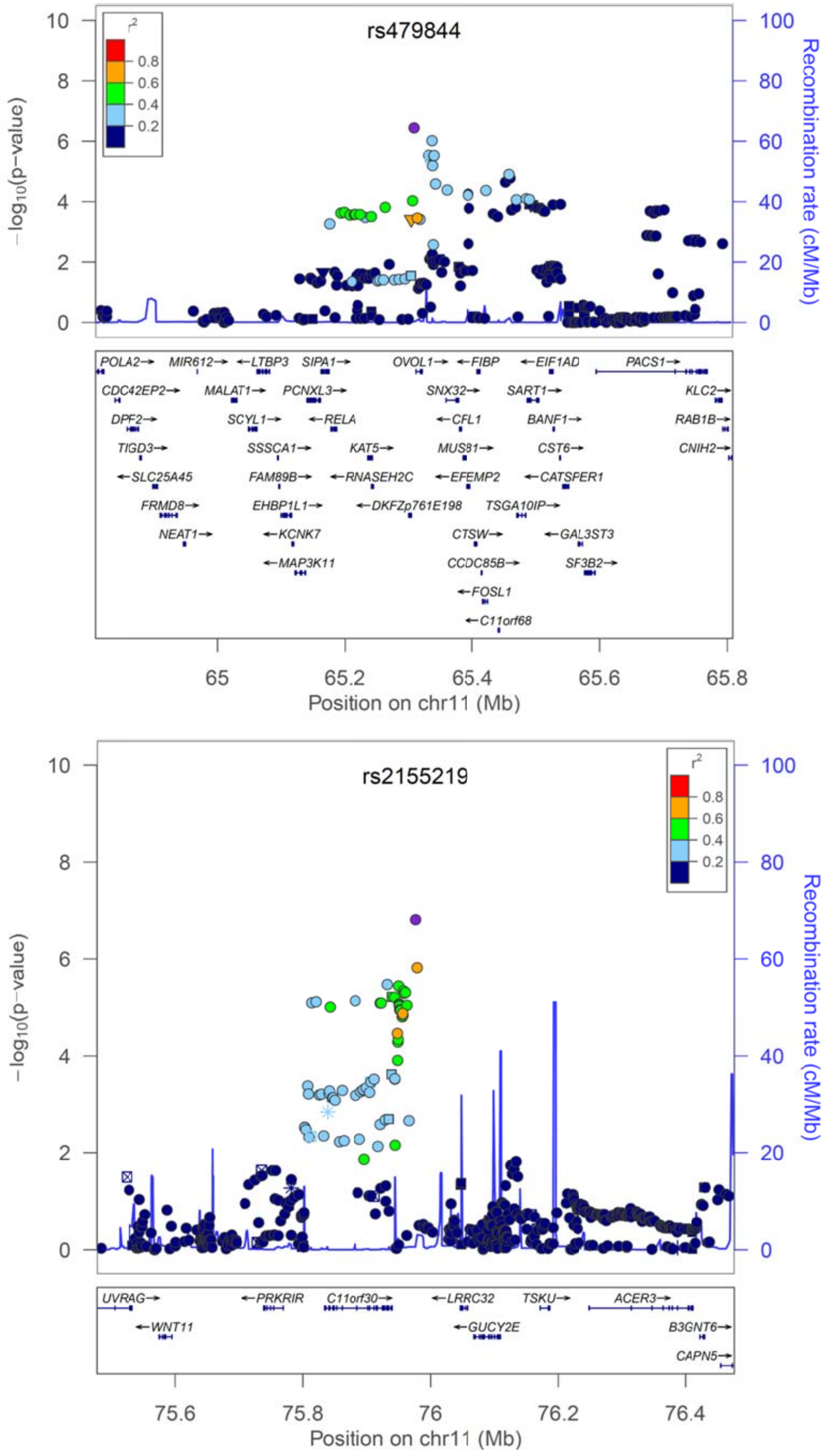
rs479844
rs2155219
−log10(p−value)
Recombination rate (cM/Mb)
Position on chr11 (Mb)
r2
POLA2 MIR612 LTBP3 SIPA1 OVOL1 FIBP EIF1AD PACS1
CDC42EP2 MALAT1 PCNXL3 SNX32 SART1 KLC2
DPF2 SCYL1 RELA CFL1 BANF1 RAB1B
TIGD3 SSSCA1 KAT5 MUS81 CST6 CNIH2
SLC25A45 FAM89B RNASEH2C EFEMP2 CATSPER1
FRMD8 EHBP1L1 DKFZp761E198 TSGA10IP
NEAT1 KCNK7 CTSW GAL3ST3
MAP3K11 CCDC85B SF3B2
FOSL1
C11orf68
UVRAG PRKRIR C11orf30 LRRC32 TSKU ACER3
WNT11 GUCY2E B3GNT6
CAPN5

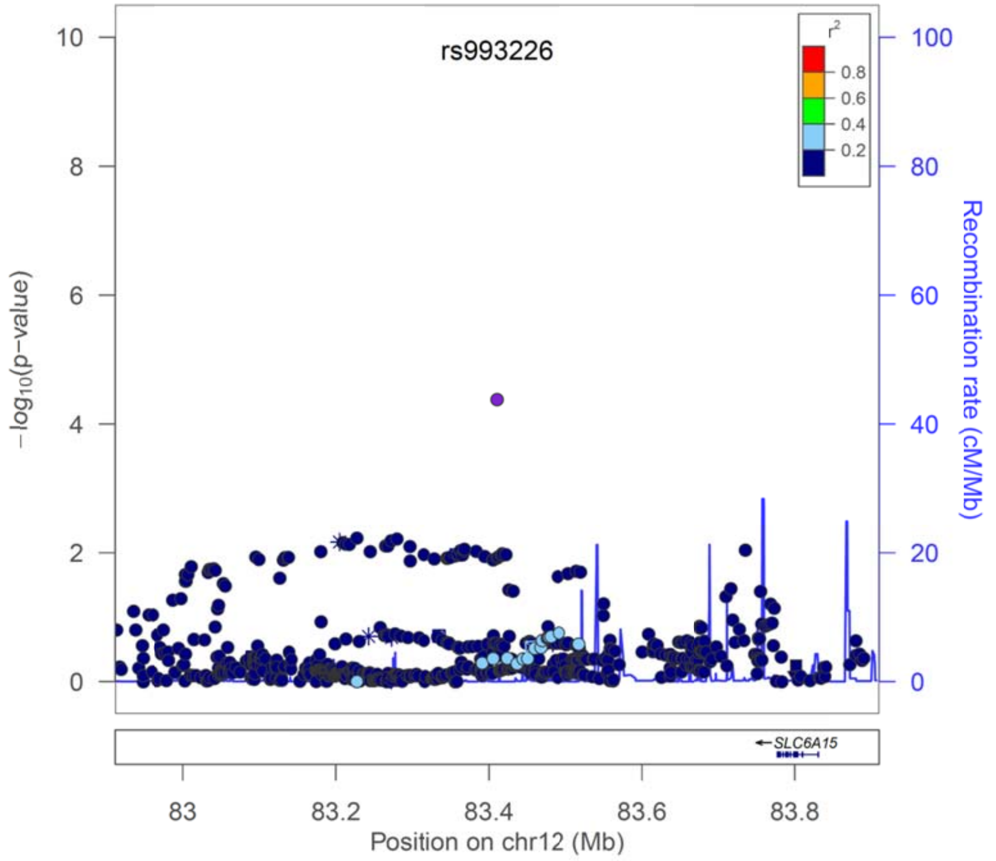
rs993226
r²
0.8
0.6
0.4
0.2
−log10(p−value)
Recombination rate (cM/Mb)
SLC6A15
Position on chr12 (Mb)

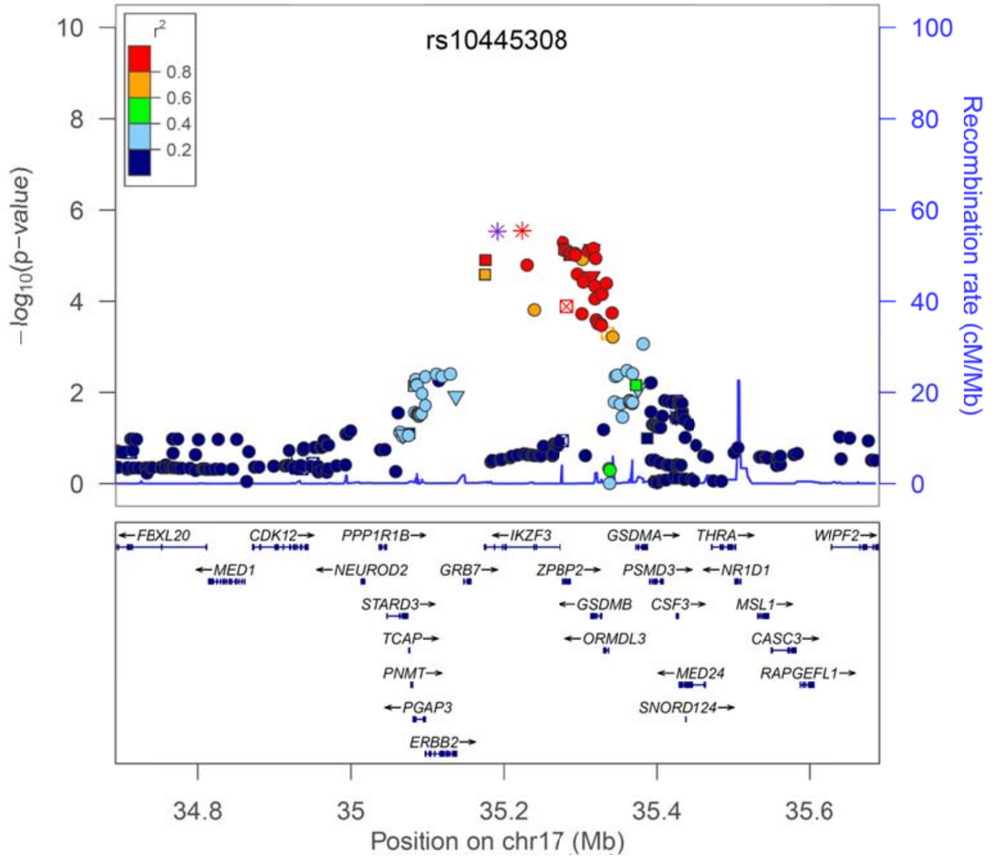
rs10445308
r²
0.8
0.6
0.4
0.2
−log10(p−value)
Recombination rate (cM/Mb)
FBXL20
CDK12
PPP1R1B
IKZF3
GSDMA
THRA
WIPF2
MED1
NEUROD2
GRB7
ZPBP2
PSMD3
NR1D1
STARD3
GSDMB
CSF3
MSL1
TCAP
ORMDL3
CASC3
PNMT
MED24
RAPGEFL1
PGAP3
SNORD124
ERBB2
Position on chr17 (Mb)

Supplementary Figure 4. Detailed results of the regions associated with the atopic march. A 1-Mb window around each lead SNP (purple) is shown. Genomic position (x-axis) including the annotated genes and *P* values (y-axis) of the SNPs under study (dots) for the discovery meta-analysis are indicated. Dots are colored according to the extent of LD (measured by r^2) between the respective SNP and the lead SNP in the region. Blue peaks represent recombination rates (y-axis). Plots were generated with LocusZoom (http://csg.sph.umich.edu/locuszoom/).[1]

rs17690965

Study	Allele frequency	R^2 / ProperInfo
ALSPAC	0.244	0.993
B58C	0.239	0.990
BAMSE	0.298	0.977
EGEA	0.274	0.953
German GWAS	0.279	0.968
PIAMA	0.271	0.992
Australian GWAS	0.249	0.934
CHOP	0.260	0.994
GENEVA	0.272	0.991
MAGICS	0.299	0.983
MAS	0.277	0.988
**Summary**	**0.264**	

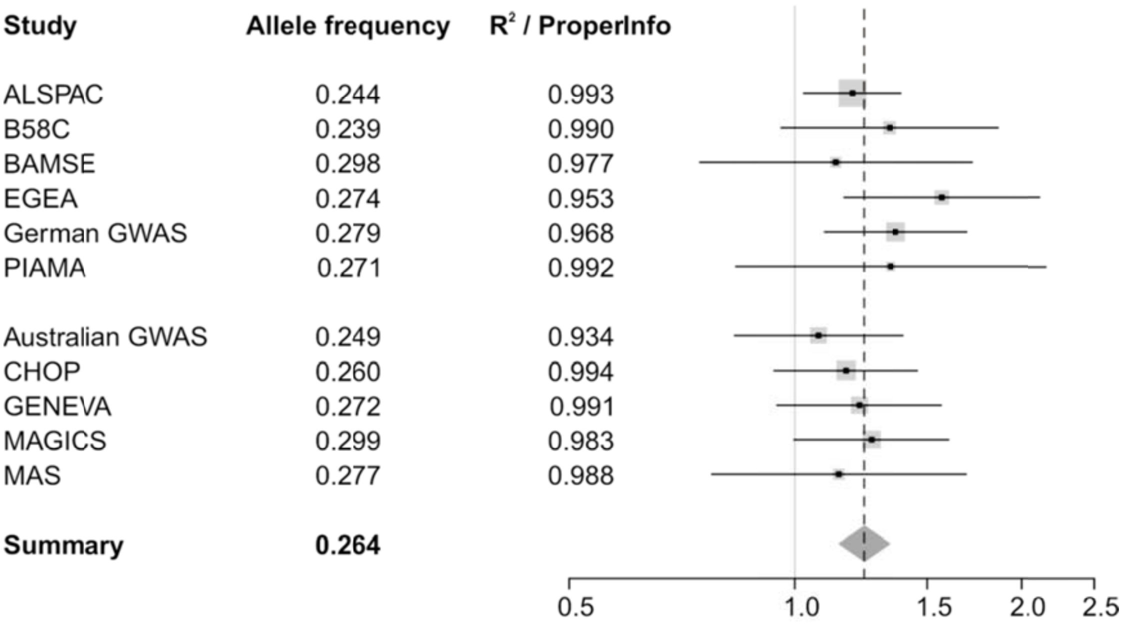

rs9357733

Study	Allele frequency	R^2 / ProperInfo
ALSPAC	0.817	1
B58C	0.807	1
BAMSE	0.795	0.999
EGEA	0.832	0.978
German GWAS	0.807	0.959
PIAMA	0.856	0.992
Australian GWAS	0.808	0.993
CHOP	0.809	0.993
GENEVA	0.801	0.992
German Replication	0.792	1
MAGICS	0.810	0.968
MAS	0.809	1
**Summary**	**0.808**	

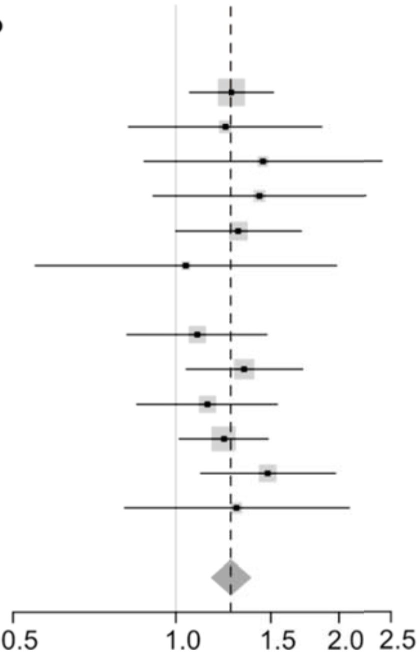

rs479844

Study	Allele frequency	R^2 / ProperInfo
ALSPAC	0.444	1
B58C	0.444	0.999
BAMSE	0.483	0.999
EGEA	0.438	1
German GWAS	0.420	0.965
PIAMA	0.497	1
Australian GWAS	0.450	1
CHOP	0.444	1
GENEVA	0.433	1
MAGICS	0.414	0.997
MAS	0.444	1
**Summary**	**0.44**	

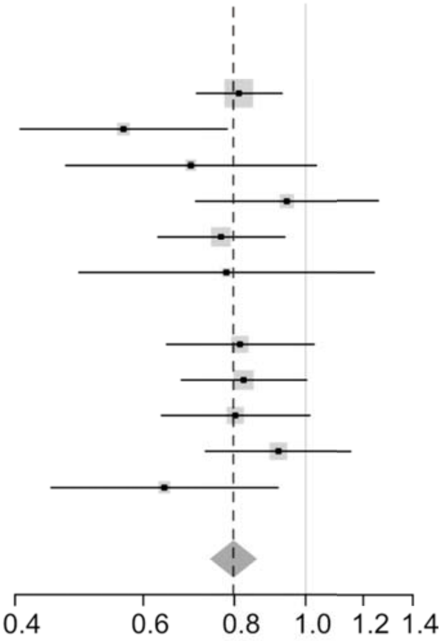

rs2155219

Study	Allele frequency	R^2 / ProperInfo
ALSPAC	0.484	0.923
B58C	0.476	0.939
BAMSE	0.414	0.938
EGEA	0.505	0.919
German GWAS	0.479	0.961
PIAMA	0.484	0.932
Australian GWAS	0.487	0.917
CHOP	0.493	0.948
GENEVA	0.497	0.956
MAGICS	0.497	0.919
MAS	0.500	0.927
**Summary**	**0.487**	

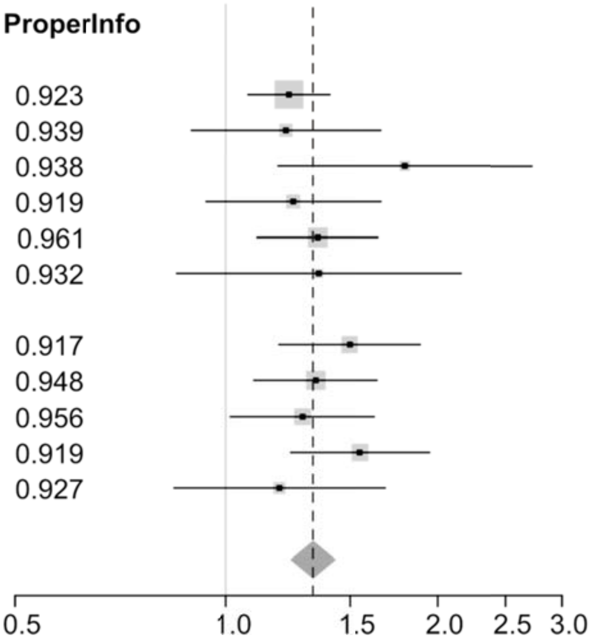

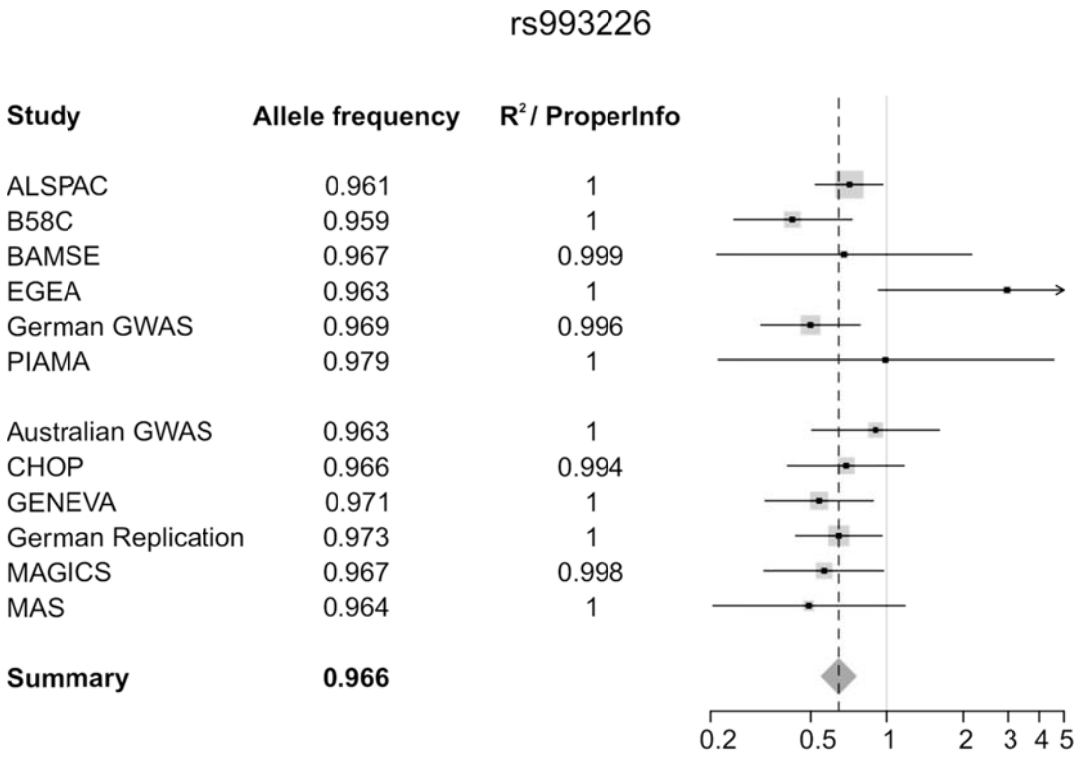
rs993226
Study
Allele frequency
R² / ProperInfo
ALSPAC 0.961 1
B58C 0.959 1
BAMSE 0.967 0.999
EGEA 0.963 1
German GWAS 0.969 0.996
PIAMA 0.979 1
Australian GWAS 0.963 1
CHOP 0.966 0.994
GENEVA 0.971 1
German Replication 0.973 1
MAGICS 0.967 0.998
MAS 0.964 1
Summary 0.966
0.2 0.5 1 2 3 4 5

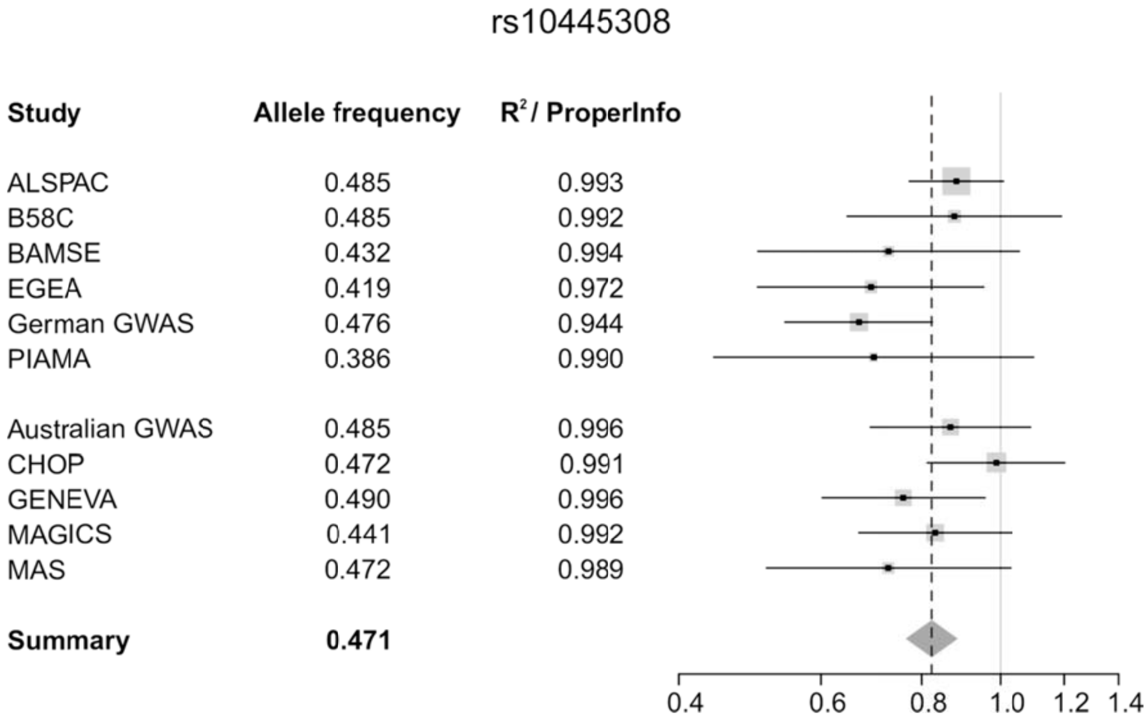
rs10445308
Study
Allele frequency
R² / ProperInfo
ALSPAC 0.485 0.993
B58C 0.485 0.992
BAMSE 0.432 0.994
EGEA 0.419 0.972
German GWAS 0.476 0.944
PIAMA 0.386 0.990
Australian GWAS 0.485 0.996
CHOP 0.472 0.991
GENEVA 0.490 0.996
MAGICS 0.441 0.992
MAS 0.472 0.989
Summary 0.471
0.4 0.6 0.8 1.0 1.2 1.4

Supplementary Figure 5. Effect sizes of the SNPs associated with the atopic march in all study populations of the discovery phase and of the replication phase. Forest plots show odds ratios (black dots) and 95% confidence intervals (bars) for the risk alleles (Table 1) in each study population. Risk allele frequencies and imputation quality are indicated. Grey boxes represent the weight of each individual study in the meta-analysis, the diamond represents the overall summary estimate. Forest plots were generated with R using the rmeta package.

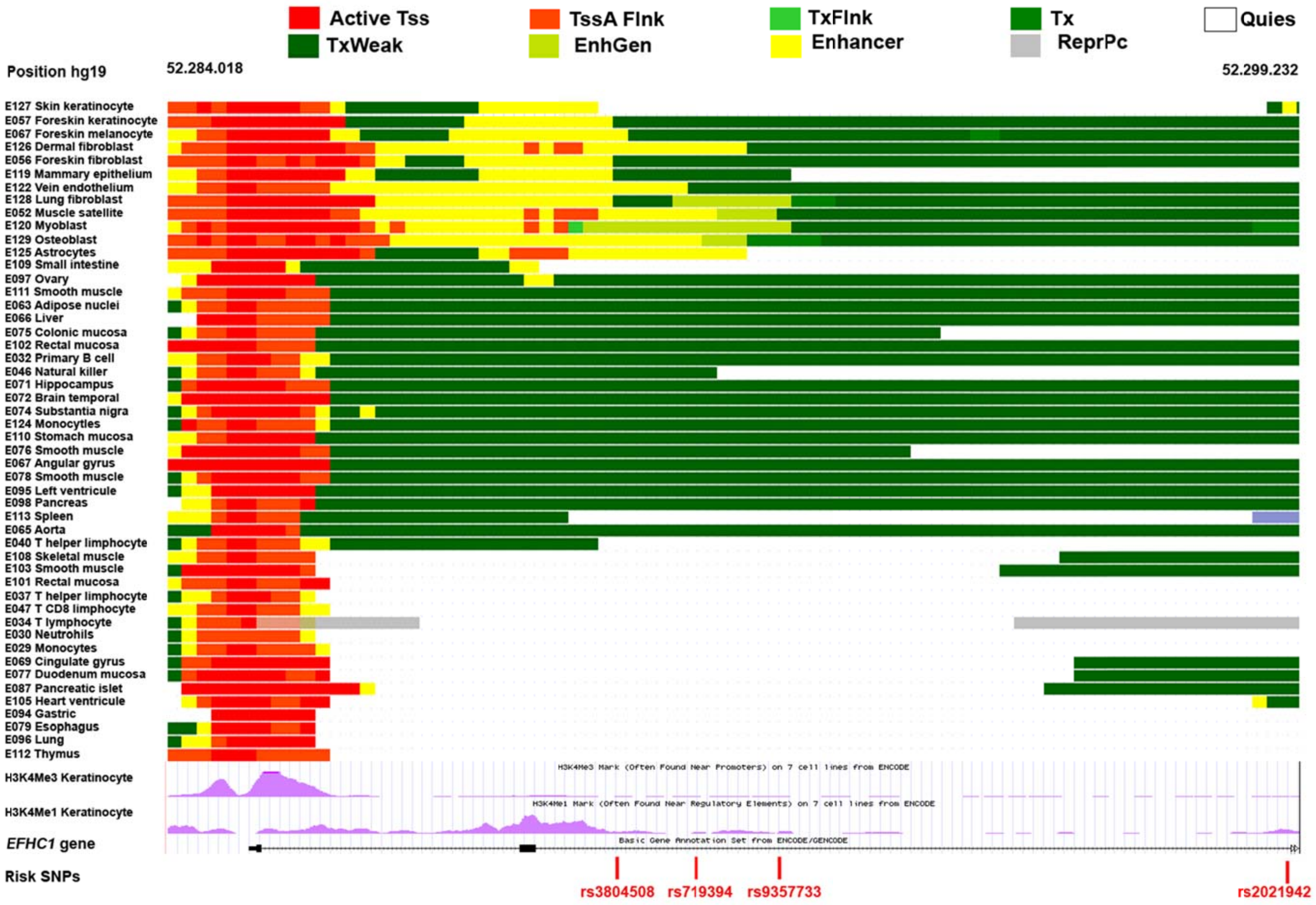

Active Tss
TxWeak
TssA Flnk
EnhGen
TxFlnk
Enhancer
Tx
ReprPc
Quies
Position hg19
52.284.018
52.299.232
E127 Skin keratinocyte
E057 Foreskin keratinocyte
E067 Foreskin melanocyte
E126 Dermal fibroblast
E056 Foreskin fibroblast
E119 Mammary epithelium
E122 Vein endothelium
E128 Lung fibroblast
E052 Muscle satellite
E120 Myoblast
E129 Osteoblast
E125 Astrocytes
E109 Small intestine
E097 Ovary
E111 Smooth muscle
E063 Adipose nuclei
E066 Liver
E075 Colonic mucosa
E102 Rectal mucosa
E032 Primary B cell
E046 Natural killer
E071 Hippocampus
E072 Brain temporal
E074 Substantia nigra
E124 Monocytles
E110 Stomach mucosa
E076 Smooth muscle
E067 Angular gyrus
E078 Smooth muscle
E095 Left ventricule
E098 Pancreas
E113 Spleen
E065 Aorta
E040 T helper limphocyte
E108 Skeletal muscle
E103 Smooth muscle
E101 Rectal mucosa
E037 T helper limphocyte
E047 T CD8 limphocyte
E034 T lymphocyte
E030 Neutrohils
E029 Monocytes
E069 Cingulate gyrus
E077 Duodenum mucosa
E087 Pancreatic islet
E105 Heart ventricule
E094 Gastric
E079 Esophagus
E096 Lung
E112 Thymus
H3K4Me3 Keratinocyte
H3K4Me3 Mark (Often Found Near Promoters) on 7 cell lines from ENCODE
H3K4Me1 Keratinocyte
H3K4Me1 Mark (Often Found Near Regulatory Elements) on 7 cell lines from ENCODE
EFHC1 gene
Basic Gene Annotation Set from ENCODE/GENCODE
Risk SNPs
rs3804508
rs719394
rs9357733
rs2021942

Supplementary Figure 6. Enhancer marks within the new atopic march susceptibility locus on chromosome 6p12.3. Snapshot of the UCSC browser showing predicted enhancer co-localizing with risk SNPs in the region (http://genomebrowser.wustl.edu/). In the upper part we show the primaryHMM segmentation track from the NIH Roadmap Epigenomics Consortium, which used five histone marks to predict functional states in the human genome.[2] The functional states were defined as Active Tss = Active transcriptional start site, TssAFlnk = Flanking active TSS, TxFlnk = Transcription at gene 5' and 3' regions, Tx = Strong transcription, Tx weak = Weak transcription, EnhG = Genic enhancers, Enhancer = Transcriptional Enhancer, ReprePC = Repressed Polycomb, Quies = Quiescent/low. All cells from primary tissues with a predicted enhancer (yellow) are displayed. Additionally, a selection of tissues and organs without evidence for an enhancer in that region is shown. In the middle part, the ENCODE layered H3K4Me1 and layered H3K4Me3 tracks for normal human epidermal keratinocytes are shown. The bottom part indicates all SNPs in LD (r^2 > 0.8) with the risk variant rs9357733 as identified using 1000G data on the SNAP server.[3]

Supplementary Table 1. Characterization of the study populations.

Study population[a]	Country	No of cases, strict definition[b] (% males)	No of cases, wide definition[b] (% males)	No of controls (% males)	Mean age (cases, strict /cases, wide / controls)	Genotyping platform
**Discovery phase**						
ALSPAC	UK	554 (57%)	745 (56.1%)	2266 (51.6%)	13.9/13.9/13.9	Illumina Human Hap 550-quad
B58C	UK	86 (67.4%)	147 (55.1%)	4829 (51.3%)	16.0/16.0/16.0	Illumina 550Kv1, 550Kv3 or 610K
BAMSE	Sweden	75 (65%)	110 (61%)	228 (47%)	12.3/12.3/12.3	Illumina 610K quad
EGEA	France	102 (63%)	189 (58%)	843 (48%)	10.5/17.4/35.6	Illumina 610K quad
German GWAS	Germany	238 (58%)	266 (57%)	1792 (50.1%)	11.6/11.7/50.0	Affymetrix 500K or 6.0
PIAMA	Netherlands	96 (63.5%)	120 (61.7%)	72 (45.8%)	8.1/8.1/8.0	Illumina 610K quad
GABRIELA	Germany	n.a.	314 (65%)	690 (51%)	n.a./8.8/8.7	Illumina 610K quad
SLSJ	Canada	n.a.	128 (45%)	137 (56%)	n.a./19.8/49.3	Illumina 610K quad
TOMSK	Russia	n.a.	56 (59%)	334 (55%)	n.a./9.6/39.2	Illumina 610K quad
**Replication 1**						
Australian GWAS	Australia	160 (46.9%)	363 (45.5%)	1958 (40.6%)	16.1/29.1/41.0	Illumina 610K quad
CHOP	USA	247 (70.0%)	433 (63.0%)	1233 (51.5%)	2.6/4.3/8.4	Illumina HumanHap550K, 610K quad or OmniExpress
GENEVA/POPGEN/KORA	Germany	164 (47.6%)	174 (47.1%)	1247 (47.9%)	24.9/25.4/50.8	Illumina HumanHap300K or 550K
MAGICS/ISAAC	Germany	214 (62.1%)	269 (62.5%)	529 (49.0%)	10.6/10.6/9.6	Illumina HumanHap300K
MAS/HNR	Germany	79 (70%)	103 (68%)	379 (47.8%)	13.0/13.0/54.7	Illumina 610K quad / HumanHap550K
RAINE	Australia	n.a.	249 (85.1%)	590 (51.7)	n.a./17.1/17.0	Illumina 660K
**Replication 2**						
German Replication	Germany	413 (59.3)	413 (59.3)	1658 (50.3%)	9.1/9.1/50.0	TaqMan / Affymetrix 6.0

[a] For a detailed description of the study populations see Methods.

[b] Study populations fulfilling the strict phenotype definition of the atopic march (see Methods) are shaded grey. A total of 4 study populations had no early data on eczema available, they were analyzed for the wide definition of the atopic march; n.a., not available.

Supplementary Table 2. Phenotype definitions of the study populations.

Study population	Asthma definition	Eczema definition (early onset)	Eczema definition (childhood onset)	Controls
Discovery set				
ALSPAC	Doctor's diagnosis of asthma ever (parental report at age 91, 128, or 166 months)	2 or more parental reports of "rash in the previous 12 months" at age 6, 18, 30, or 42 months plus a doctor's diagnosis ever of eczema or a doctor's diagnosis ever of eczema unknown (parental reports at ages 128 and 166 months)	Early eczema phenotype or a doctor's diagnosis ever of eczema (parental reports at age 128 or 166 months)	No doctor's diagnosis ever of asthma (parental reports at ages 91, 128, and 166 months), no doctor's diagnosis ever of eczema (parental reports at 128 and 166 months) and no parental report of rash in the previous 12 months (at ages 6, 18, 30, and 42 months); at least one negative report each on asthma and on eczema/rash
B58C	Parental report of asthma or wheezy bronchitis ever or in the last 12 months at age 7, 11, or 16 years	Parental report of eczema in the first year of life at 7 years follow-up	Parental report of eczema ever at age 7 yrs or of eczema in the last 12 months at age 11 or 16 years or visible eczema at school examinations	No parental report of asthma/wheezy bronchitis and of eczema ever and no visible eczema at school examinations (at age 7, 11, and 16 years)
BAMSE	Doctor's diagnosis of asthma ever (parental report at age 1, 2, 4, 8, or 12 years)	Doctor's diagnosis of eczema ever (parental report at age 1 or 2 years)	Doctor's diagnosis of eczema ever (parental report at age 1, 2, 4, 8, or 12 years)	No doctor's diagnosis of asthma and of eczema ever (parental reports at ages 1, 2, 4, 8, and 12 years)
EGEA	Self-report or parental report of asthma attacks ever or of attacks of breathlessness at rest with wheezing ever with age of onset up to age 16 years	Parental report of eczema in the first 2 years of life	Self-report/parental report of eczema in childhood ever (up to age 16 years)	No report of asthma and of eczema up to age 16 years
German GWAS	Doctor's diagnosis of asthma with age of onset up to age 16 years (clinical examination by a study clinician)	Doctor's diagnosis of eczema with age of onset up to age 3 years (clinical examination by a study clinician)	Doctor's diagnosis of eczema with age of onset up to age 16 years (clinical examination by a study clinician)	Population-based

Study population	Asthma definition	Eczema definition (early onset)	Eczema definition (childhood onset)	Controls
PIAMA	Doctor's diagnosis of asthma ever (parental report at age 8 years)	Doctor's diagnosis of eczema ever (parental report at age 2 years) or in the last 12 months (parental report at age 3 years) or a report of itchy rash on the flexural sites, around the ears or the eyes, or on the front of the ankles in the last 12 months (at age 1, 2, or 3 years)	Doctor's diagnosis of eczema ever (parental report at age 2 years) or in the last 12 months (parental report at age 3, 4, 5, 6, 7, or 8 years) or a report of itchy rash on the flexural sites, around the ears or the eyes, or on the front of the ankles in last 12 months (at age 1, 2, 3, 4, 5, 6, 7, or 8 years)	No doctor's diagnosis of asthma and eczema ever, no wheeze and itchy rash on the flexural sites, around the ears or the eyes, or on the front of the ankles ever (parental reports at ages 1, 2, 3, 4, 5, 6, 7, and 8 years)
GABRIELA	Doctor's diagnosis of asthma ever or at least two diagnoses of obstructive bronchitis (parental report up to age 13 years)	Not available	Doctor's diagnosis of eczema ever or an itchy rash that was stronger or weaker for at least 6 months during the last 12 months (parental report up to age 13 years)	No doctor's diagnosis of asthma, obstructive bronchitis, and eczema ever, no itchy rash that was stronger or weaker for at least 6 months during the last 12 months (parental report up to age 13 years)
SLSJ	At least 3 clinic visits for acute asthma or 2 asthma-related hospital admissions or 6 months of corticosteroid use (2 out of 3 criteria within one year) or parental/self-report report plus doctor's diagnosis of asthma ever (age of onset up to age 16 years)	Not available	Self-report of current eczema or eczema ever (age of onset up to age 16 years)	No doctor's diagnosis of asthma ever and no eczema ever (parental report/self-report)
TOMSK	Doctor's diagnosis of asthma with age of onset up to age 16 years (clinical examination by a study clinician)	Not available	Doctor's diagnosis of eczema with onset up to age 16 years (clinical examination by a study clinician)	No doctor's diagnosis of asthma and eczema ever (clinical examination and parental report/self-report)
Replication sets				
Australian GWAS	Doctor's diagnosis of asthma ever up to age 16 years (parental report / self-report)	Doctor's diagnosis, parental or self-report of eczema with onset up to age 3 years	Doctor's diagnosis, parental or self-report of eczema with onset up to age 16 years	No doctor's diagnosis of asthma and eczema ever (parental report / self-report)

Study population	Asthma definition	Eczema definition (early onset)	Eczema definition (childhood onset)	Controls
CHOP	Doctor's diagnosis of asthma with age of onset up to age 16 years (ICD9 code 493 in clinical records)	Doctor's diagnosis of eczema with age of onset up to age 3 years (ICD9 code 691.8 in clinical records)	Doctor's diagnosis of eczema with age of onset up to age 16 years (ICD9 code 691.8 in clinical records)	No doctor's diagnosis of asthma and eczema ever (clinical records)
GENEVA/ POPGEN/ KORA	Doctor's diagnosis of asthma with age of onset up to age 16 years (parental report / self-report)	Doctor's diagnosis of eczema with age of onset up to age 3 years (clinical examination and parental report / self-report)	Doctor's diagnosis of eczema with age of onset up to age 16 years (clinical examination and parental report / self-report)	Population-based
German Replication	Doctor's diagnosis of asthma with age of onset up to age 16 years (clinical examination by a study clinician)	Doctor's diagnosis of eczema with age of onset up to age 3 years (clinical examination by a study clinician)	Doctor's diagnosis of eczema with age of onset up to age 16 years (clinical examination by a study clinician)	Population-based
MAGICS / ISAAC	Doctor's diagnosis of asthma with age of onset up to age 16 years (clinical examination by a study clinician)	Doctor's diagnosis of eczema with age of onset up to age 3 years (parental report)	Doctor's diagnosis of eczema with age of onset up to age 16 years (parental report)	No doctor's diagnosis ever of asthma and eczema (parental report)
MAS / HNR	Parental report ever of asthma or wheezing or whistling in the chest in the last 12 months (from age 6 to age 13 years)	Doctor's diagnosis or parental report ever of eczema in the last 12 months (from birth to age 3 years)	Doctor's diagnosis or parental report ever of eczema in the last 12 months (from birth to age 13 years)	Population-based
Raine	Doctor's diagnosis ever of asthma (parental report / self-report at age 5, 8, 10, 14, or 16 years)	Not available	Doctor's diagnosis ever of eczema (parental report / self-report at age 5, 14, or 16 years)	No report of asthma and of eczema up to age 16 years

Supplementary Table 3. Association results of the SNPs selected for replication.

					Discovery phase				Replication phase 1				D+R1 combined			
SNP ID	Chr	Position[a]	EA	AA	AF	OR	95% CI	*P* value	AF	OR	95% CI	*P* value	AF	OR	95% CI	*P* value
rs6697238	1	30415317	c	t	0.063	1.55	(1.28-1.85)	6.5×10^{-6}	0.061	1.06	(0.83-1.37)	0.61	0.062	1.35	(1.16-1.57)	9.4×10^{-5}
rs12081541[b]	1	150707990	c	t	0.091	1.61	(1.41-1.89)	8.5×10^{-11}	n.a.	n.a.	n.a.	n.a.	n.a.	n.a.	n.a.	n.a.
rs7528235	1	227228002	t	c	0.105	1.34	(1.16-1.54)	9.4×10^{-5}	0.115	1.00	(0.83-1.20)	0.98	0.109	1.19	(1.06-1.34)	2.4×10^{-3}
rs10206423	2	39970659	t	c	0.013	2.02	(1.46-2.77)	1.7×10^{-5}	0.013	1.45	(0.86-2.45)	0.16	0.013	1.84	(1.40-2.42)	1.1×10^{-5}
rs12105600	2	64576015	c	a	0.417	1.22	(1.11-1.33)	2.9×10^{-5}	0.417	0.98	(0.88-1.09)	0.66	0.417	1.11	(1.03-1.19)	4.2×10^{-3}
rs10208708[c]	2	102131873	c	g	0.827	1.33	(1.16-1.51)	2.3×10^{-5}	0.809	1.26	(1.04-1.52)	0.097	0.821	1.3	(1.17-1.45)	1.4×10^{-6}
rs855047	2	108196665	t	c	0.320	1.24	(1.12-1.37)	2.2×10^{-5}	0.307	0.96	(0.86-1.08)	0.095	0.314	1.11	(1.03-1.20)	5.6×10^{-3}
rs12465526	2	113416452	g	a	0.258	1.24	(1.12-1.37)	3.4×10^{-5}	0.256	1.10	(0.98-1.23)	0.38	0.257	1.18	(1.09-1.27)	2.4×10^{-5}
rs641780	2	140800540	c	g	0.479	1.22	(1.11-1.34)	2.0×10^{-5}	0.482	0.96	(0.86-1.06)	0.51	0.481	1.1	(1.02-1.17)	9.2×10^{-3}
rs11676084	2	169412220	a	g	0.260	1.24	(1.12-1.37)	2.7×10^{-5}	0.262	0.96	(0.84-1.09)	0.90	0.261	1.13	(1.04-1.22)	2.9×10^{-3}
rs4605332	2	184585452	a	g	0.958	1.94	(1.44-2.60)	1.1×10^{-5}	0.955	1.02	(0.78-1.32)	0.19	0.957	1.35	(1.11-1.64)	2.6×10^{-3}
rs2161837	2	185896165	g	a	0.401	1.24	(1.14-1.37)	4.8×10^{-6}	0.396	1.08	(0.96-1.19)	0.29	0.399	1.17	(1.09-1.25)	1.7×10^{-5}
rs6712400	2	196079615	a	c	0.282	1.25	(1.13-1.38)	9.7×10^{-6}	0.282	1.06	(0.95-1.19)	0.79	0.282	1.17	(1.08-1.26)	5.7×10^{-5}
rs1179657	2	217497188	t	c	0.047	1.53	(1.25-1.87)	2.9×10^{-5}	0.042	1.03	(0.80-1.33)	0.67	0.045	1.31	(1.12-1.54)	5.9×10^{-4}
rs6431470	2	237111004	c	t	0.068	1.42	(1.19-1.67)	6.5×10^{-5}	0.071	1.11	(0.91-1.37)	0.30	0.069	1.28	(1.12-1.46)	2.0×10^{-4}
rs9850756	3	18880743	t	c	0.364	1.23	(1.11-1.36)	4.5×10^{-5}	0.360	1.01	(0.89-1.14)	0.18	0.363	1.14	(1.05-1.23)	1.0×10^{-3}
rs1372550	3	19456597	a	g	0.374	1.21	(1.11-1.33)	4.1×10^{-5}	0.371	1.02	(0.91-1.13)	0.77	0.373	1.13	(1.05-1.21)	9.2×10^{-4}
rs9881814	3	44086906	c	a	0.812	1.30	(1.14-1.47)	6.0×10^{-5}	0.816	0.84	(0.72-0.97)	0.020	0.814	1.08	(0.98-1.19)	1.2×10^{-1}
rs10513495	3	157951664	g	a	0.076	1.40	(1.19-1.64)	3.0×10^{-5}	0.072	0.93	(0.76-1.14)	0.49	0.075	1.2	(1.06-1.36)	3.7×10^{-3}
rs11926331	3	180114659	c	t	0.699	1.25	(1.12-1.39)	2.7×10^{-5}	0.698	1.03	(0.92-1.15)	0.63	0.699	1.14	(1.06-1.23)	6.1×10^{-4}

					Discovery phase				Replication phase 1				D+R1 combined			
SNP ID	Chr	Position[a]	EA	AA	AF	OR	95% CI	*P* value	AF	OR	95% CI	*P* value	AF	OR	95% CI	*P* value
rs6771859	3	190642046	t	c	0.471	1.21	(1.10-1.32)	5.0×10^{-5}	0.480	0.98	(0.87-1.10)	0.68	0.474	1.11	(1.04-1.20)	3.2×10^{-3}
rs9993187	4	54849007	c	t	0.163	1.28	(1.14-1.45)	5.4×10^{-5}	0.148	0.94	(0.81-1.10)	0.43	0.157	1.13	(1.03-1.25)	8.5×10^{-3}
rs10003871	4	142479872	t	g	0.574	1.29	(1.17-1.41)	1.6×10^{-7}	0.575	1.05	(0.94-1.17)	0.36	0.575	1.18	(1.10-1.26)	6.3×10^{-6}
rs17690965	5	132058566	c	g	0.259	1.28	(1.16-1.42)	1.5×10^{-6}	0.271	1.18	(1.05-1.32)	4.8×10^{-3}	0.264	1.24	(1.15-1.33)	4.5×10^{-8}
rs188433	5	168434907	c	t	0.376	1.22	(1.11-1.33)	2.4×10^{-5}	0.348	1.06	(0.95-1.18)	0.31	0.365	1.15	(1.07-1.23)	1.0×10^{-4}
rs4960124	6	5598993	g	a	0.321	1.23	(1.11-1.37)	7.0×10^{-5}	0.320	1.11	(0.99-1.23)	0.070	0.321	1.17	(1.09-1.27)	3.4×10^{-5}
rs471942	6	33804763	t	c	0.057	1.46	(1.22-1.75)	4.2×10^{-5}	0.051	1.12	(0.88-1.42)	0.36	0.055	1.32	(1.15-1.53)	1.3×10^{-4}
rs9357733	6	52400095	a	g	0.815	1.29	(1.13-1.46)	9.5×10^{-5}	0.807	1.27	(1.11-1.45)	5.1×10^{-4}	0.812	1.28	(1.17-1.40)	1.7×10^{-7}
rs17068227	6	107885021	c	t	0.073	1.42	(1.20-1.67)	2.9×10^{-5}	0.070	1.14	(0.94-1.37)	0.18	0.072	1.29	(1.14-1.46)	5.7×10^{-5}
rs2391278	7	26452563	c	a	0.686	1.25	(1.14-1.39)	1.7×10^{-5}	0.666	1.02	(0.90-1.15)	0.78	0.678	1.15	(1.06-1.25)	4.6×10^{-4}
rs10271519	7	34509633	g	a	0.893	1.57	(1.30-1.89)	1.6×10^{-6}	0.898	1.03	(0.84-1.25)	0.79	0.895	1.29	(1.13-1.48)	2.0×10^{-4}
rs2032588	7	87017379	a	g	0.057	1.43	(1.20-1.70)	6.0×10^{-5}	0.058	1.05	(0.82-1.35)	0.68	0.057	1.29	(1.12-1.49)	4.1×10^{-4}
rs7834120	8	63185861	c	a	0.192	1.27	(1.12-1.41)	7.2×10^{-5}	0.173	0.99	(0.86-1.14)	0.88	0.185	1.14	(1.05-1.25)	3.0×10^{-3}
rs2236321	9	5352101	g	a	0.255	1.25	(1.12-1.39)	1.9×10^{-5}	0.259	0.99	(0.88-1.12)	0.90	0.257	1.13	(1.05-1.23)	1.6×10^{-3}
rs2095044	9	6182796	t	c	0.279	1.24	(1.12-1.37)	3.4×10^{-5}	0.260	1.09	(0.97-1.23)	0.13	0.271	1.17	(1.09-1.27)	3.8×10^{-5}
rs600728	9	27143866	c	t	0.077	1.40	(1.19-1.64)	3.1×10^{-5}	0.072	1.06	(0.86-1.32)	0.54	0.075	1.27	(1.12-1.44)	2.0×10^{-4}
rs2148664	9	133144682	a	g	0.592	1.23	(1.11-1.36)	4.6×10^{-5}	0.585	1.02	(0.91-1.13)	0.77	0.589	1.12	(1.05-1.21)	1.6×10^{-3}
rs1856582	10	20815437	c	a	0.135	1.32	(1.15-1.49)	5.2×10^{-5}	0.122	0.93	(0.78-1.11)	0.44	0.131	1.17	(1.05-1.30)	4.5×10^{-3}
rs2393902	10	64039007	c	t	0.315	1.22	(1.11-1.35)	4.9×10^{-5}	0.318	1.14	(1.01-1.27)	0.030	0.317	1.18	(1.10-1.27)	7.5×10^{-6}
rs4758332	11	8286416	t	c	0.689	1.28	(1.15-1.42)	3.9×10^{-6}	0.684	1.00	(0.89-1.12)	0.98	0.687	1.14	(1.06-1.24)	6.3×10^{-4}
rs479844	11	65308533	g	a	0.558	1.28	(1.16-1.41)	3.6×10^{-7}	0.563	1.22	(1.10-1.35)	2.0×10^{-4}	0.56	1.25	(1.17-1.34)	3.6×10^{-10}

					Discovery phase				Replication phase 1				D+R1 combined			
SNP ID	Chr	Position[a]	EA	AA	AF	OR	95% CI	*P* value	AF	OR	95% CI	*P* value	AF	OR	95% CI	*P* value
rs2155219	11	75976842	t	g	0.481	1.28	(1.17-1.41)	1.5×10^{-7}	0.494	1.39	(1.25-1.54)	1.2×10^{-9}	0.487	1.33	(1.24-1.43)	1.8×10^{-15}
rs12286581	11	116253954	c	t	0.058	1.46	(1.22-1.75)	3.8×10^{-5}	0.060	1.10	(0.89-1.37)	0.37	0.059	1.3	(1.13-1.49)	1.8×10^{-4}
rs993226	12	83410704	g	t	0.037	1.58	(1.27-1.96)	4.2×10^{-5}	0.033	1.59	(1.23-2.04)	4.0×10^{-4}	0.035	1.58	(1.34-1.87)	6.2×10^{-8}
rs3886480	12	105139373	a	t	0.154	1.29	(1.14-1.46)	3.6×10^{-5}	0.150	0.85	(0.73-0.99)	0.032	0.152	1.09	(0.99-1.20)	6.5×10^{-2}
rs190133	14	69474427	t	c	0.547	1.24	(1.13-1.36)	9.1×10^{-6}	0.538	0.98	(0.88-1.09)	0.72	0.543	1.12	(1.04-1.20)	2.3×10^{-3}
rs741198	14	91437164	t	c	0.059	1.49	(1.23-1.80)	4.5×10^{-5}	0.055	0.95	(0.74-1.23)	0.71	0.058	1.27	(1.09-1.47)	2.4×10^{-3}
rs13337803	16	5887893	c	t	0.161	1.29	(1.15-1.45)	1.6×10^{-5}	0.159	1.08	(0.93-1.23)	0.35	0.16	1.2	(1.09-1.31)	8.4×10^{-5}
rs10445308	17	35191573	c	t	0.530	1.25	(1.14-1.37)	2.9×10^{-6}	0.528	1.18	(1.05-1.30)	2.9×10^{-3}	0.53	1.22	(1.13-1.30)	4.7×10^{-8}
rs11878163	18	9362470	g	a	0.376	1.24	(1.12-1.37)	6.4×10^{-6}	0.365	0.95	(0.85-1.06)	0.39	0.371	1.11	(1.03-1.19)	4.7×10^{-3}
rs4806074	19	33403638	c	t	0.144	1.31	(1.16-1.47)	1.6×10^{-5}	0.147	0.93	(0.80-1.08)	0.35	0.146	1.14	(1.04-1.25)	6.3×10^{-3}
rs6128191	20	55834861	g	a	0.104	1.38	(1.19-1.59)	1.1×10^{-5}	0.108	0.83	(0.68-1.01)	0.070	0.106	1.16	(1.03-1.30)	1.3×10^{-2}
rs17759053	21	25032989	a	g	0.075	1.37	(1.17-1.60)	8.9×10^{-5}	0.078	1.07	(0.86-1.33)	0.57	0.076	1.26	(1.11-1.43)	4.2×10^{-4}
rs2836249	21	38553334	t	c	0.758	1.25	(1.12-1.40)	8.1×10^{-5}	0.750	1.03	(0.92-1.16)	0.60	0.754	1.14	(1.06-1.24)	1.2×10^{-3}
rs4819808	22	17947149	g	a	0.750	1.30	(1.16-1.45)	5.2×10^{-6}	0.768	1.06	(0.93-1.20)	0.38	0.758	1.19	(1.09-1.29)	6.4×10^{-5}

EA, effect allele; AA, alternative allele; AF, effect allele frequency; OR, odds ratio; CI, confidence interval; n.a., not analyzed

[a] Genomic positions were based on human genome reference NCBI Build 36.3.

[b] rs12081541 represents the known *FLG* risk locus and was not subjected to replication

[c] SNPs which did not fulfil the quality criteria in more than 1 replication study

Supplementary Table 4. Results for loci previously found in GWA studies on eczema.

Region[a]	Population (reference)	Reported gene	Result of the original GWAS[b]				Result in the atopic march discovery phase[c]						Replication[d]
			SNP	EA/AA	AF	OR	SNP	R^2	EA/AA	AF	OR	*P* value	
1q21.3	EUR[4]	*TCHH (FLG)*	rs9050	A/C	0.06	1.33	rs12081541	0.17	C/T	0.09	1.61	8.5×10^{-11}	++
	ASN[5]	*FLG*	rs3126085	A/G	0.58	1.22	same			0.14	1.10	0.15	
1q21.3	EUR[6]	*IL6R*	rs8192284	C/A	0.38	1.15	rs4129267	0.97	T/C	0.41	1.17	7.0×10^{-4}	++
2q12.1	ASN[7]	*IL1RL1/IL18R1/IL18RAP*	rs13015714	G/T	0.41	1.27	same			0.24	1.19	1.4×10^{-3}	+
3p22.2	ASN[7]	*GLB1*	rs7613051	A/G	0.45	1.29	same			0.22	1.05	0.44	
	ASN[7]	*GLB1*	rs6780220	C/A	0.54	1.25	same			0.15	1.05	0.49	
3q13.2	ASN[7]	*CCDC80/LOC100630917*	rs12634229	C/T	0.33	1.29	same			0.07	0.99	0.93	
4q27	EUR[8]	*IL2/IL21*	rs17389644	A/G	0.24	1.19	same			0.25	1.11	0.06	
5q22.2	ASN[5]	*TMEM232/SLCA25A46*	rs7701890	G/A	0.13	1.24	same			0.08	1.10	0.26	
5q31.1[e]	EUR[8]	RAD50/IL13	rs848	A/C	0.21	1.40	same			0.20	1.28	2.6×10^{-5}	++
5q31.1[d]	EUR[4]	*KIF3A*	rs2897442	C/T	0.29	1.11	same			0.26	1.27	5.0×10^{-6}	++
6p21.33-32	ASN[7]	*HLA-C*	rs9368677	G/A	0.8	1.36	n.a.						
	ASN[7]	*GPSM3*	rs176095	A/G	0.81	1.40	same			0.76	0.98	0.68	
7p22.3	ASN[7]	*CARD11*	rs4722404	C/T	0.33	1.18	same			0.44	1.02	0.6	
10q21.2	ASN[7]	*ZNF365*	rs10995251	C/T	0.51	1.28	same			0.63	1.05	0.27	
11p15.4	ASN[7]	*OR10A3/NLRP10*	rs878860	C/T	0.54	1.31	same			0.59	1.01	0.76	
11p12	EUR[8]	*PRR5L*	rs12295535	T/C	0.02	1.68	same			0.03	1.47	5.0×10^{-3}	+
11q13.1	EUR[4]	OVOL1	rs479844	G/A	0.56	1.14	same			0.56	1.28	3.6 x 10-7	++

Region[a]	Population (reference)	Reported gene	Result of the original GWAS[b]				Result in the atopic march discovery phase[c]						Replication[d]
			SNP	EA/AA	AF	OR	SNP	R^2	EA/AA	AF	OR	*P* value	
<u>11q13.5</u>	EUR[9]	*C11orf30*	rs7927894	T/C	0.36	1.22	same			0.39	1.26	1.5×10^{-6}	++
16p13.13	EUR[8]	*CLEC16A/DEXI*	rs2041733	T/C	0.45	1.23	same			0.44	1.12	0.01	+
17q21.32	EUR[8]	*ZNF652*	rs16948048	G/A	0.38	1.17	same			0.38	1.10	0.04	+
19p13.2	EUR[4]	*ACTL9*	rs2164983	A/C	0.15	1.16	rs2967676	0.94	C/A	0.16	1.19	8.5×10^{-3}	+
20q13.2	ASN[7]	*CYP24A1/PFDN4*	rs16999165	A/G	0.69	1.19	same			0.97	0.86	0.23	
20q13.33	ASN[5]	*TNFRSF6B/ZGPAT*	rs6010620	G/A	0.26	1.17	same			0.77	1.06	0.30	

EUR, European; ASN, Asian; n.a., not available. European studies are shaded grey.

[a] 5 loci identified in both GWAS on eczema and GWAS on asthma are underlined.

[b] All SNPs associated with eczema in previous GWAS at $P < 5 \times 10^{-8}$ are listed including effect allele (EA) and alternative allele (AA) based on the forward strand, effect allele frequency (AF), and odds ratio (OR) (according to the NHGRI GWAS catalog available at www.genome.gov/gwastudies [accessed 02/14]). In addition, new eczema loci identified in two large-scale fine-mapping/candidate gene studies, which also reached genome-wide significance, were included. Positions are reported according to NCBI Build 36.3.

[c] Results of the respective SNPs in the atopic march discovery phase are shown, including AF, OR, and *P* value. If a reported SNP was not available in our data set, we used the best proxy SNP based on pairwise LD (r^2) according to the CEU HapMap data (http://www.broadinstitute.org/mpg/snap/ldsearchpw.php).[10] Replication of the risk alleles identified in Asian populations failed in most cases likely due to the diverse genetic architecture in different ethnic groups.[11]

[d] Nominally significant association with the same risk allele is indicated for $P < 0.001$ ("++") and $P < 0.05$ ("+").

[e] For 5q31, two independent eczema susceptibility loci were reported which were both included.

Supplementary Table 5. Results for loci previously found in GWA studies on asthma.

Region[a]	Population (reference)	Subphenotype	Reported gene	Result of the original GWAS[b]				Result in the atopic march discovery phase[c]						Replication[d]
				SNP	EA/AA	AF	OR	SNP	R^2	EA/AA	AF	OR	*P* value	
1q21.3	EUR[12]		*IL6R*	rs4129267	T/C	0.37	1.09	same			0.41	1.17	7.0×10^{-4}	++
1q23.1	AFR[13]		*PYHIN1*	rs1102000	T/C	0.72	1.34	n.a.						
1q31.3	EUR[14]	Childhood onset	*DENND1B/ CRB1*	rs2786098	T/G	0.15	0.70	same			0.22	1.06	0.28	
2q12.1	EUR[15]	Severe asthma, childhood	*IL1R1*	rs1558641	G/A	0.85	1.56	same			0.84	1.30	1.0×10^{-4}	++
	EUR[16]		*IL1RL1/ IL18R1*	rs13408661	G/A	0.84	1.23	same			0.87	1.28	7.9×10^{-4}	++
	MIXED[13]		*IL1RL1*	rs10173081	G/T	0.74-0.89	1.20	same			0.87	1.28	7.8×10^{-4}	++
	EUR[17]	Asthma and hay fever	*IL1RL1*	rs10197862	A/G	0.85-0.86	1.24	same			0.86	1.26	1.2×10^{-3}	+
	EUR[18]		*IL18R1*	rs3771166	G/A	0.62	1.15	same			0.63	1.14	0.01	+
3p26.2	EUR[19]	Childhood onset	*IL5RA*	rs9815663	T/C	0.18-0.21	0.66-0.84	same			0.20	0.95	0.41	
4p14	EUR[17]	Asthma and hay fever	*TLR1*	rs4833095	T/C	0.74-0.76	1.20	rs2101521	0.95	G/A	0.79	1.10	0.11	
4q31.21	ASN[20]		*LOC729675*	rs7686660	T/G	0.27	1.16	same			0.75	0.92	0.12	
5q12.1	EUR[21]		*PDE4D*	rs1588265	G/A	0.29	1.18	same			0.31	1.00	0.95	
5q22.1	ASN[22]		*TSLP*	rs1837253	C/T	0.35	1.17	same			0.74	1.14	0.03	+
	MIXED[13]			same	C/T	0.69-0.74	1.19							
	EUR[17]	Asthma and hay fever		same	C/T	0.71-0.75	1.17							
	EUR[17]	Asthma and hay fever	*WDR36/ CAMK4*	rs1438673	C/T	0.49-0.52	1.16	same			0.51	1.09	0.06	
5q31.1	EUR[15]	Severe asthma, childhood	*RAD50*	rs6871536	C/T	0.22	1.44	same			0.21	1.22	5.4×10^{-4}	++

Region[a]	Population (reference)	Subphenotype	Reported gene	Result of the original GWAS[b]				Result in the atopic march discovery phase[c]						Replication[d]
				SNP	EA/AA	AF	OR	SNP	R^2	EA/AA	AF	OR	*P* value	
6p21.33-32	ASN[20]		*PBX2*	rs204993	A/G	0.58	1.17	same			0.71	0.93	0.16	
	ASN[20]		*NOTCH4*	rs404860	T/C	0.50	1.21	same			0.83	1.04	0.49	
	ASN[20]		*C6orf10*	rs3129943	A/G	0.62	1.17	same			0.74	1.02	0.66	
	ASN[20]		*BTNL2*	rs3117098	G/A	0.25	1.16	same			0.31	0.96	0.48	
	EUR[16]		*BTNL2/ HLA-DRA*	rs9268516	T/C	0.24	1.15	rs9268499	0.98	A/G	0.32	1.08	0.14	
	ASN[20]		*HLA-DRA*	rs3129890	T/C	0.61	1.15	rs9268977	0.82	T/C	0.82	1.11	0.10	
	EUR[22]		*HLA-DQA1*	rs9272346	G/A	n.a.	0.93	same			0.40	0.86	2.9×10^{-3}	+
	EUR[18]		*HLA-DQ*	rs9273349	C/T	0.58	1.18	rs9272346	0.58					
	EUR[17]	Asthma and hay fever	*HLA-DQB1*	rs9273373	G/A	0.54-0.58	1.24	rs9272346	0.70					
	ASN[20]		*HLA-DQB1*	rs7775228	T/C	0.63	1.17	same			0.87	0.98	0.80	
	ASN[20]		*HLA-DQA2*	rs9275698	A/G	0.79	1.18	same			0.65	0.98	0.70	
	ASN[20]		*HLA-DOA*	rs9500927	A/G	0.26	1.13	n.a.						
	ASN[23]	Childhood onset	*HLA-DPB1*	rs987870	G/A	0.14	1.40	rs2071354	1.00	C/T	0.14	1.11	0.13	
7q22.3	EUR[15]	Severe asthma, childhood	*CDHR3*	rs6967330	A/G	0.19	1.45	rs10488047	0.62	T/C	0.12	1.15	0.05	
8q21.13	EUR[17]	Asthma and hay fever	*ZBTB10*	rs7009110	T/C	0.36-0.41	1.14	same			0.39	1.08	0.12	
8q24.11	ASN[23]	Childhood onset	*SLC30A8*	rs3019885	G/T	0.31	1.34	same			0.45	1.00	1	
9p24.1	EUR[17]	Asthma and hay fever	*IL33*	rs72699186	T/A	0.15-0.16	1.26	rs1342326	1.00	C/A	0.17	1.22	7.4×10^{-4}	++
	EUR[18]		*IL33*	rs1342326	C/A	0.16	1.20	same			0.17	1.22	7.4×10^{-4}	++
	MIXED[13]		*IL33*	rs2381416	C/A	0.23-0.56	1.18	same			0.28	1.23	4.6×10^{-5}	++
	EUR[15]	Severe asthma,	*IL33*	rs928413	G/A	0.28	1.50	same			0.26	1.22	1.1×10^{-4}	++

Region[a]	Population (reference)	Subphenotype	Reported gene	Result of the original GWAS[b]				Result in the atopic march discovery phase[c]						Replication[d]
				SNP	EA/AA	AF	OR	SNP	R^2	EA/AA	AF	OR	*P* value	
		childhood												
10p14	ASN[20]		*LOC338591*	rs10508372	G/A	0.43	1.16	same			0.93	0.98	0.85	
11q13.5	EUR[12]		*LRRC32*	rs7130588	G/A	0.34	1.09	same			0.36	1.22	3.4×10^{-5}	++
11q24.2*	EUR[19]	Childhood onset	*n.a.*	rs7927044	A/G	0.01-0.02	0.12-0.85	n.a.						
12q13.2	ASN[20]		*CDK2*	rs2069408	G/A	0.23	1.15	same			0.33	1.03	0.53	
	ASN[20]		*IKZF4*	rs1701704	G/T	0.18	1.19	same			0.33	1.03	0.50	
15q22.33	EUR[18]		*SMAD3*	rs744910	G/A	0.49	1.12	same			0.49	1.04	0.37	
	EUR[17]	Asthma and hay fever	*SMAD3*	rs17294280	G/A	0.23-0.27	1.18	rs17293632	0.78	T/C	0.27	1.04	0.51	
16p13.13	EUR[17]	Asthma and hay fever	*CLEC16A*	rs62026376	C/T	0.72-0.74	1.17	rs7203459	1.00	T/C	0.74	1.05	0.41	
17q12-q21.1	EUR[15]	Severe asthma, childhood	*GSDMB*	rs2305480	G/A	0.60	2.28	same			0.54	1.23	7.2×10^{-6}	++
	MIXED[13]		*GSDMB*	rs11078927	C/T	0.55-0.87	1.27	same			0.54	1.23	6.9×10^{-6}	++
	EUR[24]	Childhood onset	*ORMDL3*	rs7216389	T/C	0.52	1.45	same			0.50	1.19	3.0×10^{-4}	++
	EUR[25]	Severe asthma	*ORMDL3*	rs4794820	A/G	n.a.	0.75	same			0.44	0.85	6.0×10^{-4}	++
	EUR[18]		*GSDMA*	rs3894194	A/G	0.45	1.17	same			0.45	1.14	8.3×10^{-3}	+
	EUR[17]	Asthma and hay fever	*GSDMA*	rs7212938	G/T	0.46-0.48	1.16	same			0.47	1.16	4.9×10^{-3}	+
22q12.3	EUR[18]		*IL2RB*	rs2284033	G/A	0.56	1.12	same			0.57	0.97	0.50	

EUR, European; ASN, Asian; AFR, African; n.a., not available; studies including European populations are shaded grey.

[a] 5 loci identified in both GWAS on eczema and GWAS on asthma are underlined.

* 1 asthma GWAS locus was not investigated, because there was no proxy SNP available.

[b] All SNPs associated with asthma in previous GWAS at $P < 5 \times 10^{-8}$ are listed including effect alleles (EA) and alternative alleles (AA) based on the forward strand, effect allele frequency (AF), and odds ratio (OR) (according to the NHGRI GWAS catalog available at www.genome.gov/gwastudies [accessed 02/14]). Positions are reported according to NCBI Build 36.3.

[c] Results of the respective SNPs in the atopic march discovery phase are shown, including AF, OR, and *P* value. If a reported SNP was not available in our data set, we used the best proxy SNP based on pairwise LD (r^2) according to the CEU HapMap data (http://www.broadinstitute.org/mpg/snap/ldsearchpw.php).[10] Replication of the risk alleles identified in Asian populations failed in most cases likely due to the diverse genetic architecture in different ethnic groups.[11]

[d] Nominally significant association with the same risk allele is indicated at $P < 0.001$ ("++") and $P < 0.05$ ("+").

Supplementary Table 6. Effects of SNPs associated with the atopic march on gene expression levels.

SNP ID	Chr	Position	Type	Gene	Log_{10} (*P* value)	Exon position
rs17690965	5	132058566	exon QTL	*PDLIM4*	-7.44	131634895-131635058
			exon QTL	*PDLIM4*	-7.36	131635617-131637046
			exon QTL	*PDLIM4*	-6.97	131634507-131634685
			exon QTL	*PDLIM4*	-6.43	131630056-131630137
			exon QTL	*PDLIM4*	-6.30	131626201-131626352
			gene QTL	*PDLIM4*	-8.08	
rs479844	11	65308533	exon QTL	*OVOL1*	-35.16	65319093-65321266
			exon QTL	*AP5B1*	-8.31	65299940-65304389
			exon QTL	*BANF1*	-6.23	65526628-65527117
			gene QTL	*OVOL1*	-29.88	
			gene QTL	*AP5B1*	-5.51	
rs10445308	17	35191573	exon QTL	*ORMDL3*	-46.61	35330820-35332464
			exon QTL	*ORMDL3*	-37.01	35332891-35333042
			exon QTL	*ORMDL3*	-33.57	35333809-35334004
			exon QTL	*GSBMB*	-16.46	35315626-35315764
			exon QTL	*ORMDL3*	-16.4	35337263-35337380
			exon QTL	*GSBMB*	-14.18	35315205-35315275
			exon QTL	*GSBMB*	-12.93	35315890-35316050
			exon QTL	*GSBMB*	-12.00	35314374-35314743
			exon QTL	*ORMDL3*	-11.89	35335402-35336620
			exon QTL	*GSBMB*	-9.53	35319535-35319703
			exon QTL	*GSBMB*	-5.77	35326861-35327319
			exon QTL	*GSBMB*	-5.62	35322105-35322276
			exon QTL	*MIEN1*	-5.22	35138935-35139384
			exon QTL	*GSBMB*	-5.22	35318737-35318821
			exon QTL	*ZPBP2*	-4.97	35286461-35287675
			gene QTL	*ORMDL3*	-27.84	

Expression quantitative trait loci (eQTL) data were derived from RNA sequencing of lymphoblastoid cell lines from 373 individuals of Northern and Western European ancestry (CEU).[26] Only for three of the atopic march SNPs, eQTL data were reported.

Supplementary Table 7. Case only analysis for the atopic march loci in the ALSPAC cohort.

				Effect allele frequency				Atopic march cases vs. eczema cases		Atopic march cases vs. asthma cases	
SNP	Locus	Original discovery[a]	EA	Atopic march	Eczema alone	Asthma alone	Controls	OR (95% CI)	*P*	OR (95% CI)	*P*
rs9357733	*EFHC1*	Atopic march	A	0,85	0,81	0,81	0,81	1,30 (1,04-1,64)	0,020	1,32 (1,06-1,64)	0,010
rs993226	*SLC6A15 / TMTC2*	Atopic march	G	0,052	0,030	0,029	0,037	1,77 (1,13-2,77)	0,010	1,88 (1,22-2,90)	4.5×10^{-3}
FLG combined[b]	*FLG*	Eczema	mut	0,086	0,063	0,026	0,033	1,39 (0,96-2,02)	0,080	3,64 (2,27-5,83)	7.7×10^{-8}
rs17690965	*IL4 / KIF3A*	Eczema	C	0,27	0,26	0,25	0,24	1,06 (0,87-1,28)	0,580	1,16 (0,96-1,40)	0,130
rs479844	*OVOL1*	Eczema	G	0,59	0,59	0,54	0,54	1,00 (0,84-1,19)	1,000	1,23 (1,04-1,45)	0,010
rs2155219	*C11orf30 / LRRC32*	Eczema	T	0,52	0,50	0,48	0,47	1,10 (0,93-1,29)	0,280	1,17 (1,00-1,37)	0,050
rs10445308	*IKZF3*	Asthma	C	0,54	0,49	0,57	0,51	1,22 (1,03-1,45)	0,020	0,86 (0,73-1,02)	0,080

EF, effect allele; OR, odds ratio; CI, confidence interval

[a]Allergic phenotype first associated with SNP

[b]Odds ratios for *FLG* combined were estimated based on the two most frequent disease-causing mutations *FLG* R501X and *FLG* 2282del4; mut, mutated allele

Supplementary Table 8. Genotype counts for the atopic march loci in the ALSPAC cohort.

	Eczema plus asthma	Eczema only	Asthma only	Controls
**FLG**				
wt/wt	368	347	448	1721
wt/mut	74	44	25	122
mut/mut	1	3	0	0
Frequency (mut)	8.6%	6.3%	2.6%	3.3%
**rs17690965**				
G/G	296	269	328	1328
G/C	227	191	230	825
C/C	40	34	29	138
Frequency (C)	27.3%	26.2%	24.5%	24.0%
**rs9357733**				
A/A	407	319	379	1513
A/G	139	161	189	692
G/G	17	14	19	86
Frequency (A)	84.6%	80.9%	80.7%	81.1%
**rs479844**				
G/G	199	170	174	649
G/A	270	246	288	1191
A/A	94	78	125	453
Frequency (G)	59.3%	59.3%	54.2%	54.3%
**rs2155219**				
T/T	168	126	141	526
T/G	251	239	280	1091
G/G	144	129	166	676
Frequency (T)	52.1%	49.7%	47.9%	46.7%
**rs993226**				
T/T	505	465	553	2125
T/G	57	28	34	162
G/G	1	1	0	4
Frequency (G)	5.2%	3.0%	2.9%	3.7%
**rs10445308**				
C/C	151	124	192	584
C/T	302	233	287	1151
T/T	110	137	108	556
Frequency (C)	53.6%	48.7%	57.2%	50.6%

Supplementary Table 9. Number of eczema and asthma cases in the participating studies.

Study population	Recruited through	No. of eczema cases[a] (reference)	No. of asthma cases[a] (reference)	No. of cases atopic march[b]
**Discovery phase**				
ALSPAC	Unselected	1804 (1)	607 (5)	554
B58C	Unselected	461 (1)	213 (5)	86
BAMSE	Unselected	246 (1)	239 (5)	75
EGEA	Asthma	-	482 (5)	102
German GWAS	Eczema	1468 (11)	-	238
PIAMA	Unselected	186 (1)	172 (5)	96
**Replication 1**				
Australian GWAS	Asthma	751 (1)	2669 (2)	160
CHOP	Unselected	519 (1)	793 (29)	247
GENEVA/POPGEN/KORA	Eczema	1100 (1)	-	164
MAGICS/ISAAC	Asthma	-	630 (5)	214
MAS/HNR	Unselected	-	171 (5)	79

[a]Number of eczema and asthma cases used in the referenced GWAS.
[b]Number of atopic march cases in the current study.

Supplementary Table 10. Overlap between the atopic march study and previous GWAS on eczema or asthma.

	Eczema GWAS[a] (total no. of cases)				Asthma GWAS[b] (total no. of cases)						
Overlapping study	Esparza-Gordillo et al., 2009[9] (2637)	Paternoster et al., 2011[4] (11025)	Esparza-Gordillo et al., 2013[6] (7130)	Ellinghaus et al., 2013[8] (4376)	Himes et al., 2009[21] (1504)	Moffatt et al., 2010[18] (9728)	Sleiman et al., 2010[14] (1710)	Forno et al., 2010[19] (1504)	Ferreira et al., 2011[12] (15797)	Bonnelykke et al., 2014[15] (11903)	Ferreira et al., 2014[17] (6685)
ALSPAC											
B58C											
BAMSE											
EGEA											
German GWAS											
PIAMA											
No. (proportion) of cases overlapping with the atopic march discovery set[c]	238 (0.09)	811 (0.07)	792 (0.11)	238 (0.05)	75 (0.05)	913 (0.09)	0 (0.00)	75 (0.05)	913 (0.06	736 (0.06)	554 (0.08)
Australian GWAS											
CHOP											
GENEVA/POPGEN/KORA											
MAGICS/ISAAC											
MAS/HNR											
No. (proportion) of cases overlapping with discovery set plus replication set 1[c]	238 (0.09)	1382 (0.13)	871 (0.12)	402 (0.09)	75 (0.05)	1206 (0.12)	461 (0.27)	75 (0.05)	1366 (0.09)	983 (0.08)	714 (0.11)

[a]All GWAS on eczema of European origin are indicated. In addition, two large-scale fine-mapping/candidate gene studies, which also reached genome-wide significance ($P < 5 \times 10^{-8}$) were included.

[b]All GWAS on asthma of European origin are indicated.

[c]The number (proportion) of atopic march cases derived from study populations included in the original GWAS on eczema or asthma.

Supplementary Table 11. Association results of SNPs selected for the wide definition of the atopic march.

					Discovery phase				Replication phase			
SNP ID	Chr	Position[a]	EA	AA	AF	OR	95% CI	*P* value	AF	OR	95% CI	*P* value
rs2992068	1	26148135	g	t	0.137	1.23	(1.11-1.35)	9.0×10^{-5}	0.140	0.91	(0.80-1.03)	0.12
rs594454	1	71203056	t	g	0.616	1.17	(1.09-1.26)	4.0×10^{-5}	0.629	1.01	(0.93-1.09)	0.89
rs11102703	1	114267633	c	a	0.720	1.19	(1.09-1.30)	8.5×10^{-5}	0.715	1.05	(0.96-1.15)	0.24
rs12081541[b]	1	150707990	c	t	0.093	1.49	(1.33-1.69)	3.1×10^{-11}	n.a.	n.a.	n.a.	n.a.
rs12121910*	1	186314253	t	a	0.265	1.19	(1.09-1.28)	5.0×10^{-5}	0.279	0.99	(0.89-1.09)	0.80
rs10920129	1	199376526	t	c	0.245	1.19	(1.09-1.30)	6.6×10^{-5}	0.245	0.95	(0.86-1.05)	0.34
rs10208708*[c]	2	102131873	c	g	0.827	1.24	(1.12-1.37)	4.2×10^{-5}	0.814	1.11	(0.97-1.28)	0.14
rs641780*	2	140800540	c	g	0.486	1.16	(1.08-1.25)	5.3×10^{-5}	0.480	0.93	(0.86-1.01)	0.091
rs1179657*	2	217497188	t	c	0.046	1.51	(1.28-1.78)	9.2×10^{-7}	0.041	0.98	(0.80-1.19)	0.81
rs6431470*	2	237111004	c	t	0.068	1.33	(1.16-1.54)	4.9×10^{-5}	0.073	1.11	(0.95-1.28)	0.18
rs6803483	3	158960238	a	t	0.203	1.20	(1.10-1.31)	7.0×10^{-5}	0.196	1.01	(0.90-1.12)	0.89
rs17630607	3	159872554	t	c	0.221	1.20	(1.10-1.31)	4.0×10^{-5}	0.220	0.92	(0.84-1.02)	0.11
rs11926331*	3	180114659	c	t	0.699	1.19	(1.10-1.30)	2.9×10^{-5}	0.700	1.02	(0.93-1.11)	0.72
rs7667913	4	38550882	a	t	0.278	1.18	(1.09-1.28)	5.5×10^{-5}	0.279	1.10	(1.00-1.21)	0.051
rs9993187*	4	54849007	c	t	0.160	1.23	(1.12-1.37)	2.5×10^{-5}	0.151	0.96	(0.86-1.09)	0.53
rs10003871*	4	142479872	t	g	0.575	1.18	(1.09-1.27)	1.6×10^{-5}	0.575	1.05	(0.97-1.14)	0.25
rs4692893	4	172729618	c	t	0.354	1.18	(1.09-1.28)	3.1×10^{-5}	0.355	1.00	(0.92-1.09)	0.96
rs12659961	5	110214903	c	t	0.167	1.23	(1.11-1.37)	3.2×10^{-5}	0.178	1.10	(0.99-1.22)	0.082
rs2244012	5	131929124	g	a	0.217	1.23	(1.14-1.35)	2.3×10^{-6}	0.221	1.20	(1.10-1.33)	8.2×10^{-5}
rs188433*	5	168434907	c	t	0.367	1.18	(1.09-1.27)	3.0×10^{-5}	0.351	0.97	(0.89-1.05)	0.44

					Discovery phase				Replication phase			
SNP ID	Chr	Position[a]	EA	AA	AF	OR	95% CI	*P* value	AF	OR	95% CI	*P* value
rs404305	6	15064962	t	c	0.268	1.20	(1.11-1.31)	8.7×10^{-6}	0.257	0.94	(0.86-1.03)	0.21
rs13215347	6	25472460	g	a	0.631	1.18	(1.09-1.27)	4.8×10^{-5}	0.634	1.02	(0.94-1.11)	0.55
rs2856437	6	32265342	a	g	0.061	1.39	(1.18-1.62)	4.7×10^{-5}	0.058	1.13	(0.93-1.37)	0.23
rs2499742	6	34022238	c	t	0.599	1.18	(1.09-1.27)	7.9×10^{-5}	0.612	1.02	(0.93-1.12)	0.60
rs2126341	6	89182743	a	g	0.261	1.19	(1.09-1.30)	5.1×10^{-5}	0.264	0.97	(0.88-1.08)	0.60
rs17068227*	6	107885021	c	t	0.073	1.30	(1.14-1.49)	9.8×10^{-5}	0.073	1.15	(0.99-1.32)	0.071
rs2391278*	7	26452563	c	a	0.679	1.18	(1.09-1.28)	8.9×10^{-5}	0.668	1.03	(0.93-1.14)	0.52
rs2032588*	7	87017379	a	g	0.056	1.36	(1.17-1.57)	3.8×10^{-5}	0.057	1.01	(0.84-1.22)	0.89
rs12667496	7	114685164	t	c	0.552	1.17	(1.09-1.26)	2.7×10^{-5}	0.557	0.98	(0.90-1.06)	0.63
rs1345934	7	136422815	g	a	0.251	1.19	(1.10-1.30)	2.9×10^{-5}	0.249	1.06	(0.97-1.16)	0.16
rs7834120*	8	63185861	c	a	0.189	1.23	(1.14-1.37)	5.2×10^{-6}	0.173	1.01	(0.91-1.12)	0.87
rs4430075	8	68880814	a	g	0.664	1.18	(1.08-1.27)	9.2×10^{-5}	0.660	1.04	(0.95-1.14)	0.40
rs13269784	8	143640577	c	t	0.527	1.18	(1.09-1.27)	3.2×10^{-5}	0.526	1.02	(0.94-1.11)	0.59
rs2236321*	9	5352101	g	a	0.256	1.19	(1.10-1.30)	3.1×10^{-5}	0.261	0.98	(0.90-1.08)	0.72
rs2095044*	9	6182796	t	c	0.281	1.18	(1.09-1.28)	4.1×10^{-5}	0.263	1.06	(0.96-1.16)	0.24
rs666831	10	6012681	c	a	0.865	1.28	(1.14-1.43)	3.7×10^{-5}	0.860	0.96	(0.86-1.09)	0.53
rs440563	10	132177611	c	t	0.091	1.33	(1.18-1.52)	6.9×10^{-6}	0.093	1.10	(0.94-1.27)	0.22
rs4758332*	11	8286416	t	c	0.691	1.21	(1.11-1.31)	1.1×10^{-5}	0.686	1.01	(0.92-1.10)	0.87
rs2632065	11	19503844	c	t	0.778	1.23	(1.12-1.35)	9.9×10^{-6}	0.785	0.93	(0.85-1.03)	0.15
rs479844*	11	65308533	g	a	0.562	1.22	(1.12-1.32)	3.4×10^{-7}	0.562	1.20	(1.11-1.30)	7.6×10^{-6}
rs2155219*	11	75976842	t	g	0.483	1.25	(1.16-1.34)	1.0×10^{-8}	0.492	1.30	(1.20-1.41)	3.3×10^{-10}

					Discovery phase				Replication phase			
SNP ID	Chr	Position[a]	EA	AA	AF	OR	95% CI	*P* value	AF	OR	95% CI	*P* value
rs797191	13	32674390	c	t	0.389	1.16	(1.08-1.25)	7.3 x 10^{-5}	0.389	0.93	(0.85-1.01)	0.087
rs2352908	14	48507681	t	g	0.152	1.23	(1.12-1.36)	4.2 x 10^{-5}	0.150	0.98	(0.88-1.10)	0.77
rs10445308*	17	35191573	c	t	0.542	1.23	(1.14-1.32)	9.3 x 10^{-8}	0.533	1.19	(1.10-1.28)	2.0 x 10^{-5}
rs1893452	18	45737506	a	g	0.614	1.18	(1.10-1.28)	2.3 x 10^{-5}	0.613	1.05	(0.97-1.15)	0.22
rs1342425	20	6066729	c	g	0.318	1.18	(1.09-1.28)	3.9 x 10^{-5}	0.309	0.98	(0.90-1.07)	0.72
rs6060403	20	29684765	c	a	0.748	1.22	(1.12-1.33)	7.0 x 10^{-6}	0.758	1.00	(0.91-1.10)	0.98
rs2836249*	21	38553334	t	c	0.754	1.21	(1.10-1.32)	2.9 x 10^{-5}	0.754	1.02	(0.93-1.11)	0.75

EA, effect allele; AA, alternative allele; AF, effect allele frequency; OR, odds ratio; CI, confidence interval; n.a., not analyzed

[a] Genomic positions were based on human genome reference NCBI Build 36.3.

[b] rs12081541 represents the known *FLG* risk locus and was not subjected to replication

[c] SNPs which did not fulfil the quality criteria in more than 1 replication study

* SNPs also included in the strict atopic march analysis

Supplementary Notes

Acknowledgments

We thank William Cookson, Miriam Moffatt, Erika v. Mutius, Florence Demenais, and Ivo Gut for planning and coordinating the GABRIEL project which provided the main genotyping resource of this study and which initiated a great network of study populations aiming to analyze the molecular mechanisms underlying allergic disease.

ALSPAC: We are extremely grateful to all the families who took part in this study, the midwives for their help in recruiting them, and the whole ALSPAC team, which includes interviewers, computer and laboratory technicians, clerical workers, research scientists, volunteers, managers, receptionists and nurses. The UK Medical Research Council and the Wellcome Trust (Grant ref: 102215/2/13/2) and the University of Bristol provide core support for ALSPAC. GWAS data was generated by Sample Logistics and Genotyping Facilities at the Wellcome Trust Sanger Institute and LabCorp (Laboratory Corportation of America) using support from 23andMe.

Australian GWAS: We would like to thank all participants of the asthma studies conducted by the QIMR Berghofer Medical Research Institute (QIMR), Lung Institute of Western Australia (LIWA), Childhood Asthma Prevention Study (CAPS), Busselton Health Study, Melbourne Study of Childhood Asthma (MESCA), Tasmanian Health Study (TAHS). We would also like to thank the following AAGC collaborators: Jennie Hui, Peter Le Souëf, Patrick Danoy, Dale R Nyholt, Mark Jenkins, Catherine Hayden, Wei Ang, John Beilby, Faang Cheah, Désirée Mészáros, Mary Roberts, Melissa C Southey, Euan R Tovey, Nicole M Warrington, Loren Price, Margaret J Wright, Scott D Gordon, Li P Chung, Anjali K Henders, Graham Giles, Paul S Thomas, Suzanna Temple, John B Whitfield, Ian Feather, Stephen Morrison, Peter D Sly, Chalermchai Mitrpant, Warwick J Britton, David John, Pat G Holt, Andrew S Kemp, Pamela A Madden, Andrew C Heath, John L Hopper, Peter M Visscher, Stephen R Leeder, Craig Pennell, Haydn Walters, Graham Jones, Michael J Abramson, Shyamali C Dharmage and Matthew A Brown.

The Australian GWAS was funded by a grant from the National Health and Medical Research Council of Australia (513627).

BAMSE: We would like to thank all the participants. BAMSE was supported by The Swedish Research Council, The Swedish Heart-Lung Foundation, Stockholm County Council (ALF), the Strategic Research Programme (SFO) in Epidemiology at Karolinska Institute and the European Commission (018996).

B58C: We acknowledge use of phenotype and genotype data from the British 1958 Birth Cohort DNA collection, funded by the Medical Research Council grant G0000934 and the Wellcome Trust grant

068545/Z/02. (http://www.b58cgene.sgul.ac.uk/). Genotyping for the B58C-WTCCC subset was funded by the Wellcome Trust grant 076113/B/04/Z. The B58C-T1DGC genotyping utilized resources provided by the Type 1 Diabetes Genetics Consortium, a collaborative clinical study sponsored by the National Institute of Diabetes and Digestive and Kidney Diseases (NIDDK), National Institute of Allergy and Infectious Diseases (NIAID), National Human Genome Research Institute (NHGRI), National Institute of Child Health and Human Development (NICHD), and Juvenile Diabetes Research Foundation International (JDRF) and supported by U01 DK062418. B58C-T1DGC GWAS data were deposited by the Diabetes and Inflammation Laboratory, Cambridge Institute for Medical Research (CIMR), University of Cambridge, which is funded by Juvenile Diabetes Research Foundation International, the Wellcome Trust and the National Institute for Health Research Cambridge Biomedical Research Centre; the CIMR is in receipt of a Wellcome Trust Strategic Award (079895). The B58C-GABRIEL genotyping was supported by a contract from the European Commission Framework Programme 6 (018996) and grants from the French Ministry of Research.

CHOP: The study was funded by an Institutional Development Fund to the Center for Applied Genomics at The Children´s Hospital of Philadelphia, and by 1U01HG006828-01 from the eMERGE network.

EGEA: We thank Inserm, the French Ministry of Higher Education and Research, University Paris Diderot for supporting our research and the EGEA cooperative group. EGEA cooperative group: Coordination: V Siroux (epidemiology, PI since 2013); F Demenais (genetics); I Pin (clinical aspects); R Nadif (biology); F Kauffmann (PI 1992-2012). Respiratory epidemiology: Inserm U 700, Paris: M Korobaeff (Egea1), F Neukirch (Egea1); Inserm U 707, Paris: I Annesi-Maesano (Egea1-2) ; Inserm CESP/U 1018, Villejuif: F Kauffmann, N Le Moual, R Nadif, MP Oryszczyn (Egea1-2), R Varraso ; Inserm U 823, Grenoble: V Siroux. Genetics: Inserm U 393, Paris: J Feingold ; Inserm U 946, Paris: E Bouzigon, F Demenais, MH Dizier ; CNG, Evry: I Gut (now CNAG, Barcelona, Spain), M Lathrop (now Univ McGill, Montreal, Canada). Clinical centers: Grenoble: I Pin, C Pison; Lyon: D Ecochard (Egea1), F Gormand, Y Pacheco ; Marseille: D Charpin (Egea1), D Vervloet (Egea1-2) ; Montpellier: J Bousquet ; Paris Cochin: A Lockhart (Egea1), R Matran (now in Lille) ; Paris Necker: E Paty (Egea1-2), P Scheinmann (Egea1-2) ; Paris-Trousseau: A Grimfeld (Egea1-2), J Just. Data and quality management: Inserm ex-U155 (Egea1): J Hochez ; Inserm CESP/U 1018, Villejuif: N Le Moual ; Inserm ex-U780: C Ravault (Egea1-2) ; Inserm ex-U794: N Chateigner (Egea1-2) ; Grenoble: J Quentin-Ferran (Egea1-2).

The French EGEA study was partly funded by grants from the French National Agency for Research (ANR 05-SEST-020-02/05-9-97, ANR 06-CEBS, ANR-11-BSV1-027-GWIS-AM). Genotyping of the asthma-

ascertained samples was supported by grants from the European Commission (018996), the French Ministry of Higher Education and Research.

GABRIELA: The GABRIEL Advanced Surveys were funded by the European Commission as part of GABRIEL (A multidisciplinary study to identify the genetic and environmental causes of asthma in the European Community) contract number 018996 under the Integrated Program LSH-2004-1.2.5-1 Post genomic approaches to understand the molecular basis of asthma aiming at a preventive or therapeutic control.

GENEVA / POPGEN / KORA: The project received infrastructure support through the DFG Cluster of Excellence "Inflammation at Interfaces" (grants EXC306 and EXC306/2), and was supported by the German Federal Ministry of Education and Research (BMBF) within the framework of the e:Med research and funding concept (sysINFLAME, grant # 01ZX1306A) and the PopGen 2.0 network (01EY1103). The KORA research platform was initiated and financed by the Helmholtz Center Munich, German Research Center for Environmental Health, which is funded by the German Federal Ministry of Education and Research (BMBF) and by the State of Bavaria.

German GWAS: We thank all individuals and families for their participation in this study. We thank all physicians and nurses involved in patient recruitment for their valuable contribution to the study. We are grateful to the laboratory technicians C. Flachmeier and S. Kolberg for their work. This study was funded by Deutsche Forschungsgemeinschaft.

The SHIP authors are grateful to Mario Stanke for the opportunity to use his server cluster for SNP imputation. We thank all staff members and participants of the SHIP studies, as well as all of the genotyping staff for generating the SHIP SNP data set. SHIP is part of the Community Medicine Research net of the University of Greifswald, Germany, which is funded by the Federal Ministry of Education and Research (grants no. 01ZZ9603, 01ZZ0103, and 01ZZ0403), the Ministry of Cultural Affairs as well as the Social Ministry of the Federal State of Mecklenburg-West Pomerania, and the network ‘Greifswald Approach to Individualized Medicine (GANI_MED)’ funded by the Federal Ministry of Education and Research (grant 03IS2061A). Genome-wide data were supported by the Federal Ministry of Education and Research (grant 03ZIK012) and a joint grant from Siemens Healthcare, Erlangen, Germany, and the Federal State of Mecklenburg–West Pomerania. The University of Greifswald is a member of the 'Center of Knowledge Interchange' program of the Siemens AG and the Caché Campus program of the InterSystems GmbH.

MAGICS / ISAAC: This work was supported by the German Federal Ministry of Education and Research (BMBF) within the framework of the e:Med research and funding concept (grant # 01ZX1306E).

MAS / HNR: We wish to thank the initiators Ulrich Wahn, Renate L. Bergmann, and Karl E. Bergmann, furthermore we thank Carl-Peter Bauer, Johannes Forster, Volker Wahn, Antje Schuster, Fred Zepp, Ute Hoffmann, Christoph Grueber, Renate Krueger, Bodo Niggemann, Petra Wagner, Monika Goetze, Andreas Reich, and Gabriele Schulz. We also thank all MAS children and their parents, all collaborators and staff not listed here.

The MAS study up to adolescence was funded by several grants from the German Federal Ministry of Education and Research (07015633, 07 ALE 27, 01EE9405/5, and 01EE9406).

The collection of probands in the Heinz Nixdorf RECALL Study (HNR) was supported by the Heinz Nixdorf Foundation. The genotyping of HNR probands was financed through a grant of the German Ministry of Education and Science (BMBF).

PIAMA: We would like to thank all participating children and their families.

The PIAMA study is supported by the Dutch Asthma Foundation (grant 3.4.01.26, 3.2.06.022, 3.4.09.081 and 3.2.10.085CO), the ZonMw (a Dutch organization for health research and development; grant 912-03-031), and the ministry of the environment. Genome-wide genotyping was funded by the European Commission as part of GABRIEL (A multidisciplinary study to identify the genetic and environmental causes of asthma in the European Community) contract number 018996 under the Integrated Program LSH-2004-1.2.5-1 Post genomic approaches to understand the molecular basis of asthma aiming at a preventive or therapeutic control.

Raine: The authors would like to thank the many colleagues who contributed to collection and phenotypic characterisation of the clinical samples, as well as genotyping and analysis of the GWA data.

SLSJ: Catherine Laprise is the chairholder of the Canada Research Chair (CRC) on genetic determinants in asthma, the director of the Inflammation and Remodeling in asthma Strategic Group of the Respiratory Health Network (RHN) of the Fonds de la recherche en santé du Québec (FRSQ) and research member of AllerGen NCE Inc. (the Allergy, Genes and Environment Network).

TOMSK: The study was supported in part by Russian Foundation for Basic Research grant (01-04-48213a), INTAS grant (05-1000004-7761), and Federal Agency on Science and Innovations grant (02.434.11.7117).

Supplementary Methods

Participating studies

ALSPAC (Avon Longitudinal Study of Parents and Children; http://www.bristol.ac.uk/alspac/) is a population-based longitudinal prospective birth-cohort study which includes 14,062 children born in 1991 and 1992 in Avon, United Kingdom. Phenotype data were obtained from regular parental reports on children's health. Asthma and childhood eczema were defined based on a parental report of a doctor's diagnosis ever of asthma (at age 91, 128 or 166 months) and eczema (at age 128 or 166 months) respectively. For early eczema, at least two parental reports on skin rashes in the joints and creases of the body in the past 12 months (at age 6, 18, 30, or 42 months) were required. Children were excluded if the doctor's diagnosis contradicted the ascertainment of eczema based on early skin rashes. Controls were selected as having no doctor`s diagnoses ever of asthma and of eczema up to age 166 months and no rash ever up to age 42 months. In addition, at least one negative report each on asthma and on eczema or rash was required. A total of 9912 subjects were genotyped using the Illumina Human Hap 550-quad array (Illumina, Inc., San Diego, CA) by 23andMe as described previously.[4] Imputation was performed with MACH 1.0.16 using the HapMap 2 (release 22) CEU reference panel. Ethics approval for the study was obtained from the ALSPAC Ethics and Law Committee and the Local Research Ethics Committee.

Australian GWAS are samples from the Australian GWAS on asthma, which has been described in detail previously.[12] The study included data from 7,197 Australian individuals of European ancestry from three cohorts, Australian Asthma Genetics Consortium (AAGC), Busselton, and Queensland Institute of Medical Research studies (QIMR). Of these, 363 individuals had both a reported doctor's diagnosis of asthma and a doctor's diagnosis or a parental report/self-report of eczema up to age 16 years. For 160 cases, onset of eczema had been reported up to age 3 years. In total, 1958 controls were available who never had a diagnosis of asthma and of eczema. All samples included in our study were genotyped with the Illumina Human 610-quad array. Imputation was performed with Impute 2 using a combined 1000 Genomes Project (CEU, Mar 2010 release) and HapMap 3 (all 11 populations, Feb 2009 release) CEU reference panel. SNPs were tested for association with disease status using logistic regression in PLINK with sex included as a covariate. Participants provided informed consent to participate in this study, which was approved by the respective ethics committees.[12]

B58C (British 1958 birth cohort; http://www2.le.ac.uk/projects/birthcohort/1958BC-About) is a population-based cohort of over 17000 individuals born in 1958 in England, Scotland, or Wales. At

ages 7, 11, and 16 years, the history of asthma, wheezy bronchitis, and eczema was ascertained by parental interviews, and the presence of visible eczema was recorded at school medical examinations. Cases were defined as early onset if they had a parentally reported history of eczema in the first year of life and of asthma and/or wheezy bronchitis up to age 16 years. Childhood onset was defined as having eczema and asthma/wheezy bronchitis ever up to age 16 years. Controls were selected as having no asthma/wheezy bronchitis and no eczema up to age 16 years. DNA samples of 8,051 subjects were available. About half of them had previously been genotyped using the Illumina Human Hap 550K arrays v1 and v3 by the Wellcome Trust Sanger Institute[27] and the Type 1 Diabetes Consortium[11] respectively. All eligible asthma cases from the remaining DNA samples and a similar number of controls were genotyped by the GABRIEL consortium using the Illumina Human 610-quad array.[18] Analyses were restricted to SNPs present on all 3 arrays. Imputation was performed with MACH 1.0.16 using the HapMap 2 (release 21) CEU reference panel. Logistic regression analysis was performed with ProbAbel-0.1-3. Ethical approvals, including consent procedures, were obtained from the Southeast England Multicentre Research Ethics Committee.

BAMSE (http://snd.gu.se/en/catalogue/study/EXT0037) is a Swedish longitudinal prospective birth-cohort study of over 4000 children born between 1994 and 1996 in the area of Stockholm.[28] At ages 1, 2, 4, 8 and 12 years, parents completed questionnaires on their children’s health including allergic symptoms and diseases. Children with doctor’s diagnoses ever of asthma and of eczema as reported by the parents were defined as cases. Data on eczema up to age 2 years and up to age 12 years were used to define early onset and childhood onset of eczema respectively. Children with no history of asthma and eczema up to age 12 years were selected as controls. Genotyping and imputation was performed as part of the GABRIEL consortium.[18] Out of 2033 subjects with DNA available, all children with a doctor’s diagnoses ever of asthma and children without any allergic disease were genotyped. For genotyping, the Illumina Human 610-quad array (Illumina, Inc., San Diego, CA) was used. Imputation was accomplished with MACH 1.0.16 using the HapMap 2 (release 22) CEU reference panel. Logistic regression analysis was performed with ProbAbel. The study was approved by the Ethics Committee of Karolinska Institutet, Stockholm, Sweden.

CHOP (Children’s Hospital of Philadelphia) patients and controls were recruited at the Children’s Hospital of Philadelphia between 2006 and 2010. All subjects were of self-reported Caucasian origin and residents in the Greater Philadelphia area. Cases were defined by the presence of the ICD9 codes for both eczema (691.8) and asthma (493) in their electronic medical records. The study included 433 patients with physician-diagnosed eczema plus asthma and age of onset up to age 16 years. Of those,

237 had the first diagnosis of eczema up to age 3 years. In total, 1233 controls without eczema and without asthma were available. All CHOP samples were genotyped on either Human Hap550, Human 610 quad, or HumanOmniExpress (Illumina, San Diego) at the Center for Applied Genomics. Imputation was carried out using Impute version 1 and the HapMap 2 (release 22) haplotypes as a reference. Statistical analyses were performed with SNPTEST. Ethical approval for this study was obtained from the Institutional Review Board of the Children's Hospital of Philadelphia.

EGEA (Epidemiological study on the Genetics and Environment of Asthma, bronchial hyperresponsiveness and atopy; https://egeanet.vjf.inserm.fr/index.php/en/) is a French longitudinal survey on asthma and allergies including a case control study and a family study, in total around 2100 subjects. The first survey took place between 1991 and 1995 and recruited 348 families ascertained by one asthmatic family member attending a chest clinic, 40 families ascertained by an asthmatic sib pair, and 415 population-based controls.[29] Between 2003 and 2007, the 12-year follow-up was conducted and 58 new family members were recruited. All participants were between 7 and 70 years old at the time of the study. Phenotype data were collected through face-to-face interviews (adults) or parental interviews (children up to age 16 years) and examinations including standardized questionnaires on detailed clinical data, skin prick tests, lung function tests, and blood samples. Asthma cases were defined based on a report of asthma or breathlessness at rest with wheezing and a reported age of onset up to 16 years. A parental report of eczema of the child before 2 years of age was used for the early onset phenotype. Children with a parental report of eczema up to age 16 years and adults with self-reported eczema in childhood were defined as cases for the childhood onset study. In controls, the absence of asthma and eczema was required up to age 16 years. Genotyping and imputation of the EGEA samples was carried out within the GABRIEL consortium using the Illumina Human 610-quad array and the MACH 1.0 software with the HapMap 2 (release 22) reference panel.[18] Logistic regression analyses were performed using the option cluster within families, as implemented in the Stata logit function, including the robust sandwich estimator of the variance to account for familial dependence. Informative principal components for within-Europe diversity were included as covariates in the model. Written informed consent was obtained from all subjects (or their parents) participating in the study which was approved by the Comités de Protection des Personnes, Hôpital Cochin Royal, Paris.

GABRIELA (GABRIEL Advanced Survey) are cross-sectional population-based surveys conducted in rural areas of Austria, Germany, and Switzerland.[30] In total, 132,366 children aged 6 to 13 years were addressed through schools. In a first stage in fall/winter 2006, asthma, allergic disease, and contact

to farming environments were assessed using a short parental questionnaire (n=79,888). In a second stage in spring/summer 2007, 9,668 children were selected among families consenting in writing to blood sampling, genetic testing and collection of environmental samples by stratified random sampling to ensure representation of children with high exposure to farming environments. Asthma was defined as a parental report of a doctor's diagnosis ever of asthma or a reported diagnosis of obstructive bronchitis at least twice. Eczema was defined as a doctor's diagnosis ever of eczema or an itchy rash that was stronger or weaker for at least 6 months during the last 12 months as reported by the parents. Children without a doctor's diagnosis ever of asthma, obstructive bronchitis, eczema, and without itchy rash were selected as controls. Genomic DNA and questionnaire data were available for 7,303 children of whom 1,727 samples were genotyped within the GABRIEL consortium using the Illumina Human 610-quad array.[18] SNPs were imputed with MACH using the HapMap 2 (release 22) CEU reference panel. Stratified weighted logistic regression analyses were calculated with the Stata software. To account for the stratified random sampling, probability weights were introduced in the statistical analyses. Informed consent has been provided by the parents and the local ethics committees approved the study.[30]

GENEVA / POPGEN / KORA is a German case-control study population. GENEVA (Genetic evaluation of atopic dermatitis) comprises 1100 eczema cases of self-reported German ethnicity recruited at the Department of Dermatology and Allergy, Technical University Munich.[4] Eczema was diagnosed on the basis of a skin examination by experienced dermatologists according to standard criteria in the presence of a chronic or chronically relapsing pruritic dermatitis with the typical morphology and distribution.[31] Ascertainment of asthma was based on a parental report/self-report of a doctor's diagnosis of asthma. Age at examination or a parental report/self-report of age of onset was used to define early onset and childhood onset of eczema. Controls were obtained from the population-based studies popgen (Christian-Albrechts-University of Kiel) and KORA (Cooperative Health Research in the Region of Augsburg).[32] Only samples genotyped on either Illumina Human Hap300 or Human Hap550 array were used. In total, 164 and 174 cases with age of onset up to age 3 and 16 years respectively were included in our study as were 1247 controls fulfilling the quality criteria. Imputation was carried out with IMPUTE version 2 using the HapMap 2 (release 22) CEU reference panel. The study has been approved by the local ethics committees and informed consent has been given by all participants.[4]

German GWAS is an eczema case-control study population from Germany. Cases are tertiary care patients from Berlin, Kiel, or Munich and participated in the German GWAS on eczema.[9] Population-

based controls are from the German SHIP (Study of Health in Pomerania) study.[33] The SHIP set was split for two case-control studies (German GWAS and German Replication) by a random function. 1792 unrelated individuals were included in German GWAS. Early onset of eczema was defined according to a clinician's diagnosis of eczema up to age 3 years. Childhood onset was defined based on a clinician's diagnosis of eczema either up to age 16 years or later if onset was reported up to age 16 years. Out of 1468 subjects with eczema genotyped genome-wide previously,[9] 266 had a doctor's diagnosis of asthma up to age 16 years and were included in the discovery set (German GWAS). Genotyping was performed using the Affymetrix Human 500K or the Affymetrix Human 6.0 array. SNPs were imputed with the MACH 1.0 software using the HapMap 2 (release 22) CEU reference panel. Logistic regression analyses was performed with the Stata software including the robust sandwich estimator of the variance to account for familial dependence. The study has been approved by the local ethics committees and informed consent has been given by all participants or their legal guardians.[9] The SHIP study was approved by the ethics committee of the University of Greifswald.

German Replication

For replication, 413 unrelated patients, recruited at Charité University Medical Center Berlin, were genotyped using TaqMan Allelic discrimination. Early onset of eczema was defined according to a clinician's diagnosis of eczema up to age 3 years. Childhood onset of asthma was defined based on a clinician's diagnosis up to age 16 years or later if onset was reported up to age 16 years. Cases were analyzed with 1658 independent controls from SHIP using PLINK. The study was approved by the institutional review board of Charité University Medical Center Berlin, Berlin, Germany, and by the ethics committee of the University of Greifswald.

MAGICS / ISAAC is a German case-control study population, including children with asthma plus eczema from the Multicentre Asthma Genetics in Childhood Study (MAGICS) and German controls from the cross-sectional International Study of Asthma and Allergies in Childhood (ISAAC). Asthma cases from MAGICS were diagnosed by a paediatric pulmonologist or allergologist on the basis of clinical examination, case history, and objective tests of lung function. Between 2001 and 2007, MAGICS recruited 835 children with doctor's diagnosed asthma, of whom 728 were previously genotyped.[18] Of those, 214 and 269 children had a history of eczema with onset up to age 3 and 16 years respectively, as reported by the parents. Out of 800 German children randomly drawn from the German ISAAC population previously, all 529 subjects who had genotypes available and who had no history of asthma and eczema according to standardized questionnaires were used as controls. All samples were genotyped with the Illumina Human Hap300 array. Imputation was carried out with

MACH using the HapMap 2 (release 22) CEU reference panel. Logistic regression analysis was performed in PLINK version-1.07. Ethical approval for both MAGICS/ ISAAC was obtained from the ethics committee of the Bavarian Medical council.

MAS / HNR is a German case-control study population, including children with asthma plus eczema from the Multicenter Allergy Study (MAS) and German controls from the population-based Heinz Nixdorf Recall Study (HNR). In 1990, the MAS birth cohort recruited 1314 newborns, which were regularly followed-up.[34,35] Data were collected from examinations and questionnaires at birth, at 1, 3, 6, 12, and 18 months, and yearly from age 2 to age 13. Asthma was defined on basis of a parental report ever of asthma or of wheezing or whistling in the chest within the last 12 month from age 6 to 13 years. Eczema was defined on basis of a parental report ever of eczema within the last 12 months up to age 13 years. Controls were recruited from the population-based Heinz Nixdorf Recall study.[36] All MAS cases and HNR controls were genotyped on Illumina Human Hap610 quad and Human Hap550 arrays respectively.[18] Imputation was performed with MACH using the 1000Genome/GIANT reference panel. SNPs were tested for association with disease status using MACH2DAT. The study has been approved by the institutional review board of Charité University Medical Center Berlin, Berlin, Germany. Informed consent has been given by all participants.

PIAMA (Prevention and Incidence of Asthma and Mite Allergy; http://piama.iras.uu.nl/index-en.php) is a population-based birth cohort study consisting of 3,963 children who were born in 1996 and 1997 after recruitment through prenatal clinics in the northern, middle and southwestern part of the Netherlands.[37,38] Phenotype data were collected through parental questionnaires and 3 rounds of medical examinations. Follow-up of the children took place at age 3 months and yearly from age 1 to 8 years. Asthma cases were defined based on a doctor's diagnosis ever of asthma up to age 8 years as reported by the parents. Cases with childhood onset eczema had a doctor's diagnosis ever of eczema as reported by the parents or a positive history of an itchy rash on the flexural sites, around the ears or the eyes, or on the front of the ankles up to age 8 years. For the early onset phenotype, all reports of eczema or rash up to age 3 years were used. Controls were required to have no history of asthma, wheeze, eczema, and itchy rash at flexural sites up to age 8 years. DNA was collected from 2162 children, of whom 426 children were genotyped within the framework of the GABRIEL Consortium using the Illumina Human 610-quad array.[18] SNPs were imputed with IMPUTE version 2 using the HapMap 2 (release 22) CEU reference panel. Genome-wide association analyses were performed using SNPTEST version 1.1.5. The Medical Ethical Committees of the participating institutes approved the study.[37]

Raine (http://www.rainestudy.org.au), the Western Australian pregnancy cohort, is a longitudinal birth cohort that recruited, between 1989 and 1991, 2,900 pregnant women prior to 18-weeks gestation and regularly assessed offspring up to 21 years of age.[39] Children have been comprehensively phenotyped at birth, at ages 1, 2, 3, 5, 8, 10, 14, 16 to 17, and 21 years by trained members the Raine research team. Data collection included questionnaires completed by the child's primary carer and by the adolescent from age 14, physical assessments by trained assessors at all follow-up years, DNA collection from year 14 follow-up. Cases were defined as subjects having both a parental report/self-report of a physician's diagnosis (by a pediatrician, respiratory specialist, or GP) ever of asthma at least once in year 5, year 8, year 10, year 14, and year 16 follow-ups and a parental report/self-report of a physician's diagnosis (by a pediatrician or GP) ever of eczema at least once in year 5, year 14, and year 16 follow-ups. Inclusion criteria for controls required the absence of asthma and eczema across all available time points. Only individuals of European ancestry were included in the analysis. Genotyping was performed using the Illumina Human 660W-quad array and imputation was performed with MACH v1.0.16 using the CEU samples from HapMap 2 (release 22) as a reference panel. Association was calculated with MACH2DAT. The study was conducted with ethical approval of the Princess Margaret Hospital for Children Human Research Ethics Committee, and written informed consent was obtained from all mothers.

SLSJ (Saguenay-Lac-Saint-Jean) is an asthma study from Quebec, Canada, which consists of 253 French-Canadian families ascertained by asthmatic probands.[40] Probands were included in the study if they fulfilled at least two of the following criteria: 1) a minimum of three clinic visits for acute asthma within one year; 2) two or more asthma-related hospital admissions within one year; or 3) steroid dependency, as defined by either six months of oral, or one year of inhaled corticosteroid use. Families were included in the study if at least one parent was available for phenotypic assessment, at least one parent was unaffected, and all four grandparents were of French-Canadian origin. Family members were considered asthmatics if both a self-reported history of asthma and a history of physician-diagnosed asthma were available, or by clinical evaluation following a methacholine provocation test. Individuals were considered to have eczema when giving a positive answer to at least one of the two following questions: 1) Do you have eczema? or 2) Have you ever had eczema? SLSJ samples were genotyped with the Illumina 610-quad array for the GABRIEL consortium.[18] Imputation was carried out using the MACH 1.0 software with the HapMap 2 (release 22) reference panel. Logistic regression analyses were performed with the Stata software including the robust sandwich estimator of the variance to account for familial dependence. This study was

approved by the ethic committee of the Centre Universitaire Intégré de Santé et de Services Sociaux du Saguenay-Lac-Saint-Jean, and all subjects gave informed consent.

TOMSK is a population-based family study conducted by the Research Institute of Medical Genetics and Siberian State Medical University (Tomsk, Russia) from 1998 onwards.[41,42] In total, 196 families families recruited through a family member with atopic asthma were studied. All participants were Russians or of a mixed ethnic origin due to marriages between Russians and major East Slavonic populations (Ukrainians, Byelorussians). Both probands and their relatives were clinically examined to establish diagnosis of eczema[43] and asthma according to standard criteria (Global Initiative for Asthma: Global Strategy for Asthma Management and Prevention; http://www.ginasthma.org). Besides the clinical examination, laboratory and functional testing were conducted to assess common IgE levels (solid-phase immune-enzyme assay), specific sensitization (skin-prick tests), lung volumes (spirometry), and airway responsiveness (bronchoprovocative tests with methacholine). Childhood onset of asthma and eczema was defined based on the age at clinical examination (children) or on a self-reported age of onset up to age 16 years (adults). Controls had no history of asthma and of eczema ever. Out of 804 subjects, 738 were genotyped within the framework of the GABRIEL consortium using the Illumina Human 610-quad array.[18] Imputation was carried out with the MACH 1.0 software using the HapMap 2 (release 22) reference panel. Logistic regression analyses were performed with the Stata software including the robust sandwich estimator of the variance to account for familial dependence. The study was approved by the Ethics Committee of the Siberian State Medical University.

Supplementary References

1. Pruim,R.J. *et al.* LocusZoom: regional visualization of genome-wide association scan results. *Bioinformatics.* **26**, 2336-2337 (2010).

2. Kundaje,A. *et al.* Integrative analysis of 111 reference human epigenomes. *Nature* **518**, 317-330 (2015).

3. Johnson,A.D. *et al.* SNAP: a web-based tool for identification and annotation of proxy SNPs using HapMap. *Bioinformatics* **24**, 2938-2939 (2008).

4. Paternoster,L. *et al.* Meta-analysis of genome-wide association studies identifies three new risk loci for atopic dermatitis. *Nat. Genet.* **44**, 187-192 (2012).

5. Sun,L.D. *et al.* Genome-wide association study identifies two new susceptibility loci for atopic dermatitis in the Chinese Han population. *Nat. Genet.* **43**, 690-694 (2011).

6. Esparza-Gordillo,J. *et al.* A functional IL-6 receptor (IL6R) variant is a risk factor for persistent atopic dermatitis. *J. Allergy Clin. Immunol.* **132**, 371-377 (2013).

7. Hirota,T. *et al.* Genome-wide association study identifies eight new susceptibility loci for atopic dermatitis in the Japanese population. *Nat. Genet.* **44**, 1222-1226 (2012).

8. Ellinghaus,D. *et al.* High-density genotyping study identifies four new susceptibility loci for atopic dermatitis. *Nat. Genet.* **45**, 808-812 (2013).

9. Esparza-Gordillo,J. *et al.* A common variant on chromosome 11q13 is associated with atopic dermatitis. *Nat. Genet.* **41**, 596-601 (2009).

10. Johnson,A.D. *et al.* SNAP: a web-based tool for identification and annotation of proxy SNPs using HapMap. *Bioinformatics.* **24**, 2938-2939 (2008).

11. Barrett,J.C. *et al.* Genome-wide association study and meta-analysis find that over 40 loci affect risk of type 1 diabetes. *Nat. Genet.* **41**, 703-707 (2009).

12. Ferreira,M.A. *et al.* Identification of IL6R and chromosome 11q13.5 as risk loci for asthma. *Lancet* **378**, 1006-1014 (2011).

13. Torgerson,D.G. *et al.* Meta-analysis of genome-wide association studies of asthma in ethnically diverse North American populations. *Nat. Genet.* **43**, 887-892 (2011).

14. Sleiman,P.M. *et al.* Variants of DENND1B associated with asthma in children. *N. Engl. J. Med.* **362**, 36-44 (2010).

15. Bonnelykke,K. *et al.* A genome-wide association study identifies CDHR3 as a susceptibility locus for early childhood asthma with severe exacerbations. *Nat. Genet.* **46**, 51-55 (2014).

16. Ramasamy,A. *et al.* Genome-wide association studies of asthma in population-based cohorts confirm known and suggested loci and identify an additional association near HLA. *PLoS. One.* **7**, e44008 (2012).

17. Ferreira,M.A. *et al.* Genome-wide association analysis identifies 11 risk variants associated with the asthma with hay fever phenotype. *J. Allergy Clin. Immunol.* **133**, 1564-1571 (2014).

18. Moffatt,M.F. *et al.* A large-scale, consortium-based genomewide association study of asthma. *N. Engl. J. Med.* **363**, 1211-1221 (2010).

19. Forno,E. *et al.* Genome-wide association study of the age of onset of childhood asthma. *J. Allergy Clin. Immunol.* **130**, 83-90 (2012).

20. Hirota,T. *et al.* Genome-wide association study identifies three new susceptibility loci for adult asthma in the Japanese population. *Nat. Genet.* **43**, 893-896 (2011).

21. Himes,B.E. *et al.* Genome-wide association analysis identifies PDE4D as an asthma-susceptibility gene. *Am. J. Hum. Genet.* **84**, 581-593 (2009).

22. Lasky-Su,J. *et al.* HLA-DQ strikes again: genome-wide association study further confirms HLA-DQ in the diagnosis of asthma among adults. *Clin. Exp. Allergy* **42**, 1724-1733 (2012).

23. Noguchi,E. *et al.* Genome-wide association study identifies HLA-DP as a susceptibility gene for pediatric asthma in Asian populations. *PLoS. Genet.* **7**, e1002170 (2011).

24. Moffatt,M.F. *et al.* Genetic variants regulating ORMDL3 expression contribute to the risk of childhood asthma. *Nature* **448**, 470-473 (2007).

25. Wan,Y.I. *et al.* Genome-wide association study to identify genetic determinants of severe asthma. *Thorax* **67**, 762-768 (2012).

26. Lappalainen,T. *et al.* Transcriptome and genome sequencing uncovers functional variation in humans. *Nature* **501**, 506-511 (2013).

27. van Heel,D.A. *et al.* A genome-wide association study for celiac disease identifies risk variants in the region harboring IL2 and IL21. *Nat. Genet.* **39**, 827-829 (2007).

28. Wickman,M., Kull,I., Pershagen,G., & Nordvall,S.L. The BAMSE project: presentation of a prospective longitudinal birth cohort study. *Pediatr. Allergy Immunol.* **13 Suppl 15**, 11-13 (2002).

29. Kauffmann,F. *et al.* EGEA (Epidemiological study on the Genetics and Environment of Asthma, bronchial hyperresponsiveness and atopy)-- descriptive characteristics. *Clin. Exp. Allergy* **29 Suppl 4**, 17-21 (1999).

30. Genuneit,J. *et al.* The GABRIEL Advanced Surveys: study design, participation and evaluation of bias. *Paediatr. Perinat. Epidemiol.* **25**, 436-447 (2011).

31. Williams,H.C. *et al.* The U.K. Working Party's Diagnostic Criteria for Atopic Dermatitis. I. Derivation of a minimum set of discriminators for atopic dermatitis. *Br. J. Dermatol.* **131**, 383-396 (1994).

32. Holle,R., Happich,M., Lowel,H., & Wichmann,H.E. KORA--a research platform for population based health research. *Gesundheitswesen* **67 Suppl 1**, S19-S25 (2005).

33. Volzke,H. *et al.* Cohort profile: the study of health in Pomerania. *Int. J. Epidemiol.* **40**, 294-307 (2011).

34. Lau,S. *et al.* The development of childhood asthma: lessons from the German Multicentre Allergy Study (MAS). *Paediatr. Respir. Rev.* **3**, 265-272 (2002).

35. Nickel,R. *et al.* Messages from the German Multicentre Allergy Study. *Pediatr. Allergy Immunol.* **13 Suppl 15**, 7-10 (2002).

36. Schmermund,A. *et al.* Assessment of clinically silent atherosclerotic disease and established and novel risk factors for predicting myocardial infarction and cardiac death in healthy middle-aged subjects: rationale and design of the Heinz Nixdorf RECALL Study. Risk Factors, Evaluation of Coronary Calcium and Lifestyle. *Am. Heart J.* **144**, 212-218 (2002).

37. Brunekreef,B. *et al.* The prevention and incidence of asthma and mite allergy (PIAMA) birth cohort study: design and first results. *Pediatr. Allergy Immunol.* **13 Suppl 15**, 55-60 (2002).

38. Zuidgeest,M.G. *et al.* Persistence of asthma medication use in preschool children. *Respir. Med.* **102**, 1446-1451 (2008).

39. Macdonald,W., Newnham,J., Gurrin,L., & Evans,S. Effect of frequent prenatal ultrasound on birthweight: follow up at 1 year of age. Western Australian Pregnancy Cohort (Raine) Working Group. *Lancet* **348**, 482 (1996).

40. Laprise,C. The Saguenay-Lac-Saint-Jean asthma familial collection: the genetics of asthma in a young founder population. *Genes Immun.* **15**, 247-255 (2014).

41. Freidin,M.B., Puzyrev,V.P., Ogorodova,L.M., Kobiakova,O.S., & Kulmanakova,I.M. [Polymorphism of interleukins and interleukin receptor genes: population distribution and association with atopic bronchial asthma]. *Genetika* **38**, 1710-1718 (2002).

42. Freidin,M.B., Kobyakova,O.S., Ogorodova,L.M., & Puzyrev,V.P. Association of polymorphisms in the human IL4 and IL5 genes with atopic bronchial asthma and severity of the disease. *Comp Funct. Genomics* **4**, 346-350 (2003).

43. Hanifin,J.M. & Rajka,G. Diagnostic Features of Atopic Dermatitis. *Acta Derm. (Stockholm)* **92 (Suppl.)**, 44-47 (1980).
